# Frequency-aware signal image representation and graph transformer learning for bearing fault diagnosis

**DOI:** 10.3389/frai.2026.1922485

**Published:** 2026-08-26

**Authors:** Sandhya Mitta, Poornima Nedunchezhian

**Affiliations:** School of Computer Science and Engineering, Vellore Institute of Technology, Vellore, Tamil Nadu, India

**Keywords:** bearing fault diagnosis, frequency-aware representation, graph neural network, predictive maintenance, signal-to-image transformation, time-frequency analysis, transformer learning

## Abstract

**Introduction:**

Rolling bearing fault diagnosis is essential for predictive maintenance because bearing failures can cause unexpected downtime, increased maintenance costs, and safety risks. Conventional signal-domain and image-based approaches may not adequately capture both local frequency characteristics and global structural relationships among fault patterns.

**Methods:**

This study proposes a frequency-aware signal image representation and graph transformer learning (FSI-GTL) framework for bearing fault diagnosis. Raw vibration signals are transformed using short-time Fourier transform (STFT), wavelet packet transform (WPT), empirical mode decomposition (EMD), and cepstral analysis. The resulting representations are adaptively fused, and discriminative frequency-domain features are used to construct graph representations. A graph neural network (GNN) captures local spectral topology, while a Transformer encoder models long-range dependencies through self-attention.

**Results:**

The proposed framework achieved an average classification accuracy of 99.42% on the CWRU dataset. Cross-dataset evaluation on the Paderborn University bearing dataset achieved an average accuracy of 98.71%, demonstrating strong generalization across datasets. The framework also showed high precision, recall, and F1-score across the evaluated bearing fault categories.

**Discussion:**

The results demonstrate that integrating frequency-aware signal representations, graph-based structural learning, and Transformer-based global contextual modeling can effectively improve bearing fault discrimination. The proposed FSI-GTL framework provides a promising approach for intelligent condition monitoring and predictive maintenance.

## 1 Introduction

Rolling bearings are indispensable components in modern rotating machinery and play a crucial role in ensuring reliable operation across various industrial sectors, including manufacturing, transportation, aerospace, renewable energy systems, mining equipment, and power generation plants. The components mentioned above are intended to enable rotational movement with minimal frictional loss and mechanical abrasion. Yet, under dynamic loading and varying speeds over long operation times in a harsh working environment, continuously applying mechanical stress makes rolling bearings more prone to progressive degradation. Failure of rolling bearings, such as inner-race defect, outer-race defect, cracked/broken roller, and grease/cage damage, drastically degrades the performance of a system and results in the failure of equipment at unexpected times (Shahzad et al., 2026; Liang et al., 2025; Wang et al., 2025a). These failures usually lead to excessive maintenance costs, production stoppages, and significant safety risks in industrial automation with mission-critical characteristics. Thus, diagnosis of rolling bearings has received much attention as a premise of smart system maintenance and intelligent industrial asset management.

Since Industry 4.0 is developing rapidly, most industrial monitoring systems widely deploy various sensors for continuous health monitoring of machine condition, where vibration signals, acoustic signals, thermal signals, and operation parameters are considered important sources of feedback. Vibration signals have been proven to be one of the richest sources of information for bearing fault detection and diagnosis (Xiao et al., 2024; Zhou et al., 2025; Li et al., 2025; Shao et al., 2023; Wang et al., 2025b), because they are directly caused by the dynamics driven by the mechanical defects. However, industrial vibration signals often have noise, non-linearities, non-stationarities, variable speeds, and loads, which makes feature extraction and classifier training complicated when the fault signs are weak and hidden in the complicated noise and disturbances.

For traditional fault diagnosis methods, since these methods largely depend on expert knowledge, it is very necessary to engineer features and select an appropriate classifier. For bearing fault diagnosis, statistical features in the time domain, spectral features in the frequency domain, and time-frequency features have been widely applied. While these features work under controlled conditions, time-frequency or statistical features often achieve diagnosis well with experience; it depends greatly on the corresponding domain knowledge (Xia et al., 2024; Jiang et al., 2025; Luo et al., 2025; Chen et al., 2024). Because the extracted features in the time-frequency and time domains cannot describe the complicated non-linear relationship of industrial data in modern conditions. So, the intelligent fault diagnosis methods are much superior.

The deep learning paradigm has also revolutionized the field of intelligent fault diagnosis to a large extent. Deep neural networks deliver strong representation learning ability by automatically learning discriminative high-level features from the raw or transformed sensor data. Particularly interesting are recent signal-to-image transformation approaches, which enable vibration signals to be represented as a visual pattern and fed into deep learning models in the image domain. Time–frequency representation has effectively obtained a spectral decomposition that represents a non-stationary signal in terms of component frequencies, which improves the perception of fault frequency-related information, which is hard to perceive directly from the original signal. The resultant time–frequency image spectrum has significantly expedited the development and application of computer vision systems for machinery fault diagnosis and has shown promising classification performance (Tang et al., 2025; Cui et al., 2024; Zheng et al., 2025).

However, there are still some things that can be improved. Mostly, image-based diagnosis approaches use a grid structure to convert the vibration signal and focus on extracting spatial local features only. Although these methods can diagnose local fault features, the relation of different frequencies is not taken into account at the same time. Under practical operating conditions, bearing faults can affect multiple frequency bands, and diagnostic information may be distributed across the entire spectrum rather than being confined to a few manually selected frequency components. It is unwise to use the images directly, ignoring structural information of fault propagation.

Another promising direction that the problem has developed is the ability to learn the global dependencies in fault representation. Some of the subclinical bearing defects are associated with relatively weak dependencies over different scales and different frequency bands. A normal convolutional neural network architecture has to take local information at a static and fixed-sized receptive field into account to cover the neighborhood of input signals (Zheng et al., 2025). Thus, although the depth of the neural network may cause a larger receptive field, the long-range dependency cannot be effectively learned (Yang et al., 2025; Zhao et al., 2025; Shahzad et al., 2025). The key contextual information is missing to effectively distinguish between similar fault types.

In a recent study, attention-based learning models have raised awareness of the importance of capturing interactions among global features. The self-attention mechanism enables neural networks to simultaneously concentrate on interested regions while associating distant feature regions with each other, which would greatly benefit machinery fault diagnosis since the most indicative fault features may be complicated and emerge at different scales of frequencies and operational variables. Unfortunately, many common attention-based models fail to explicitly exploit the structural connections of frequency-domain patterns.

Motivated by the concerns described above, this study presents a frequency-aware signal image representation and graph transformer learning (FSI-GTL) for rolling bearing fault diagnosis. In the proposed FSI-GTL framework, a challenging issue lies in jointly combining frequency-domain signal analysis, structural correlation representation, and global context learning. First, we transfer the raw vibration signals into frequency-aware signal image representations, aiming at embedding fault-sensitive frequency-domain information. Next, we represent frequency-domain information as graph structures where each vertex represents important spectral components and an edge illustrates the correlation between the two spectral components. Structural learning of these graph structures can be undertaken through a graph neural network (GNN) to capture the local structural topology and correlation. Additionally, to exploit the global context and long-range correlation among different spectral components, a self-attention-based transformer encoder is introduced for global correlation learning. Together with structural learning in graph modeling, the proposed FSI-GTL method is believed to provide a more robust performance for the downstream fault classification task.

The primary objective of this study is to develop an intelligent fault diagnosis framework that effectively combines frequency-aware signal imaging, graph representation learning, and Transformer-based feature extraction to improve bearing health assessment under diverse operating conditions. Through this integration, the proposed framework aims to enhance diagnostic accuracy, strengthen feature discriminability, and provide a scalable solution for next-generation predictive maintenance systems in smart industrial environments.

### 1.1 Contributions

The major contributions of this study are summarized as follows:

1. A novel frequency-aware signal image representation and graph transformer learning (FSI-GTL) framework is proposed for intelligent rolling bearing fault diagnosis.
2. A frequency-aware signal-to-image transformation strategy is developed to convert raw vibration signals into informative visual representations while preserving fault-sensitive spectral characteristics.
3. A graph construction mechanism is introduced to model structural relationships among frequency-domain regions, enabling the extraction of topological fault information beyond conventional image representations.
4. A hybrid GNN and Transformer architecture is designed to jointly learn local structural dependencies and global contextual interactions for enhanced fault feature representation.
5. The proposed framework provides a unified solution that integrates signal processing, graph learning, and attention-based deep learning, thereby improving robustness under varying operating conditions.
6. Extensive experiments on benchmark bearing datasets demonstrate the effectiveness of the proposed framework for intelligent condition monitoring and predictive maintenance applications.

## 2 Literature review

### 2.1 Deep learning-based bearing fault diagnosis

Rolling bearing fault diagnosis has experienced significant advancements with the adoption of deep learning techniques. Traditional machine learning methods rely heavily on handcrafted feature extraction, which often requires substantial domain expertise and exhibits limited adaptability under varying operating conditions. Deep learning models overcome these limitations by automatically learning discriminative representations from raw sensor signals and transformed feature spaces.

CNNs have been used extensively for fault diagnosis because they have strong spatial feature extraction ability for vibration signal representations. Cui et al. (2024) proposed a triplet attention-enhanced residual tree-inspired decision network for feature learning that can solve the class imbalance problem for bearing datasets by hierarchical feature learning. Wang et al. (2025a,b) proposed a lightweight fault diagnosis method based on adaptive residual enhancing and a multi-group coordinate attention mechanism.

More recent reports continued the research into signal-dependent deep models. Zhao et al. (2025) presented an adaptive self-generated WaveletNet to dynamically extract wavelet-radial features of vibration signal for fault diagnosis. Similarly, Shahzad et al. (2025) introduced a multi-scale feature fusion scheme enhanced by entropy to capture benefits from complementary frequency information of rolling element bearing fault detection. These papers evidently disclose the essential role of sensitive feature extraction.

While these are encouraging, it is crucial to recognize that the local receptive field that a CNN-based model operates over is fundamentally a “sample,” so it is possible these models will struggle with tasks in which the diagnostic information needed is geographically far away from the other diagnostic features in the frequency space.

### 2.2 Transformer-based fault diagnosis

After the excellent performance of Transformers in computer vision and natural language processing fields, Transformer-based models have attracted considerable attention in intelligent fault diagnosis recently. The self-attention mechanism can learn global correlations between input features that cannot be learned by traditional convolutional operations.

The method of Bayesian variational transformer (BVT) was proposed to solve the problem of fault diagnosis for rotating machinery by Xiao et al. (2024). Presenting the fusion of Bayesian inference with Transformer learning to improve the generalization and error estimation over working conditions, Tang et al. (2025) provided a one-dimensional Vision Transformer with multi-scale residual convolution using raw vibration signals for effective feature extraction over time-frequency domains.

To enhance the robustness of the Transformer under a noisy environment, Luo et al. (2025) proposed the MSFA-Trans framework by incorporating a multiscale frequency attention mechanism into Transformer architectures. Experiments showed that the improved network greatly improved the fault-relevant feature extraction capability of the Transformer and showed a very strong capability for extracting fault-sensitive features when subjected to noise-corrupted vibration signals. A similar idea can also be seen from Shahzad et al. (2026)'s FATF framework. They aimed to use frequency domain information to increase the robustness of the Transformer and different machine states.

Although Transformer methods show favorable performance in capturing the long-range dependencies of all locations on one feature, intra-modal relationships between spectral sub-regions may not be directly modeled because the feature is typically represented as a sequence or an image token. So, the topological relationships prevalent among many spectral features in the frequency domain might not be well preserved.

### 2.3 Transfer learning and cross-domain fault diagnosis

It is widely acknowledged that obtaining enough labeled fault data with varying operation points under the actual industrial application context is impractical. Therefore, transfer learning is a significant research topic to enhance the generalization ability of the model across domains.

The survey of transfer learning-based intelligent fault diagnosis conducted by Chen et al. (2024) detailed the recent advances, open issues, and future directions. Their research stressed the importance of domain adaptation and knowledge transfer to achieve effective application of fault diagnosis.

Motivated by the above scheme, a federated transfer learning framework for rolling bearing fault diagnosis based on cloud-edge cooperation, proposed by Liang et al. (2025), is used to diagnose faults more accurately and keep private information private, based on distributed learning at edge devices. Similarly, a Swin Transformer-based multi-source heterogeneous information fusion framework for cross-domain fault diagnosis is presented by Li et al. (2025).

Most transfer learning approaches have been proposed for knowledge transfer and domain adaptation; however, they did not employ the structural relationship between frequency components, although transfer learning could greatly improve the generalization ability.

### 2.4 Graph neural networks for machinery fault diagnosis

GNNs are a relatively new type of neural network intended to work with non-Euclidean structures. By explicitly including correlations among data points in a data structure, in the form of a graph, GNNs are suitable for irregularly structured, non-grid data.

A frequency-aware graph contrastive learning framework was employed, compared with a multi-scale recursive graph network, to diagnose rotating machinery faults (Wang et al., 2025b). This new perspective represents spectral data as a graph structure and uses enhanced contrastive learning to distinguish the features. Verified through experiments, it was an effective method for providing structural information about frequency locations using a graph.

Similarly, Li et al. (2025) adopted a graph optimization approach with contrastive learning based on dual-scale spectral features. By integrating both spectral feature extraction and a graph optimization method, the graph optimization approach improved the robustness of bearing fault diagnosis. Additionally, Jiang et al. (2025) proposed a dual graph-driven robust representation learning approach for semi-supervised fault diagnosis by using two weakly supervised graph frameworks to learn representations from unlabeled data, and the diagnostic performance was boosted.

These studies indicated that graph learning is an efficient and appealing framework to learn the topology structure between frequency features. Typical graph models usually favor local neighborhood aggregation and perform poorly in modeling global interactions at long-distance ranges.

### 2.5 Multi-modal and multi-sensor information fusion

Many sources of information have been integrated into autonomous systems during the past several years to improve fault diagnosis. Multi-modal learning could potentially provide additional information on feature discriminating power or robustness.

Zheng et al. (2025) recommended GEFBL, in which multi-sensor information fusion was used to balance the imbalanced bearing fault datasets and could effectively fuse heterogeneous sensor signals and greatly improve classification performance. Furthermore, Yang et al. (2025) proposed a sound-vibration physical-information fusion method in which a physical constraint was built into the deep learning model, and the sound-vibration signal fusion could greatly enhance the fault diagnosis precision. Additionally, Jiang et al. proposed an assisted diagnosis approach based on a digital twin for the lack of measured fault samples. This framework utilized information from both virtual and physical systems for stronger diagnosis. We prove that information fusion methods are practical. Most methods focus on feature-level fusion and only implicitly utilize the relationship based on the graph structure and the global attention.

### 2.6 Research gap and motivation

Based on literature review, the following facts can be inferred: (1) CNN based method can do well in understanding local features, but unable to describe the long range dependencies of different regions; (2) transformer based method could describe long range interactions of features, but often fail to illustrate the structural relationships of frequency domain map; (3) GNN method could illustrate the topological dependency, and is usually sensitive to local comparison in neighborhood; (4) it is recognized in literature that many approaches are applied on the transformed vibration signal as image or sequence, however, the structural relation between frequency components has still been rarely explored.

To overcome these disadvantages, this study proposes an FSI-GTL framework. The approach converts the vibration signal into a frequency-aware image representation, constructs a graph structure from the extracted frequency features, adapts GNN approaches to capture the topological dependency of generated graphs, and uses a transformer to construct a self-attention mechanism to model the global feature correlation. By combining signal imaging, graph learning, and transformer models, the FSI-GTL framework can make a more effective diagnosis of rolling bearing fault under variable operating conditions.

## 3 Proposed frequency-aware signal image representation and graph transformer learning (FSI-GTL) framework

### 3.1 Proposed framework description

The objective of the proposed FSI-GTL framework is to develop a unified fault diagnosis architecture capable of simultaneously capturing frequency-domain characteristics, structural relationships among spectral components, and long-range contextual dependencies embedded within vibration signals. Currently, the available signals for bearing fault diagnosis utilize either the signal or time-frequency image inputs to the CNNs. CNNs can effectively learn local discriminative features; however, they do not well utilize the structural correlation across frequency domains or the global interdependence across the whole spectrum. Similarly, though Transformer architectures can effectively capture long-range dependencies, it usually represents the signal in the form of sequences or image patches rather than graph structure in the frequency domain.

To address these shortcomings, the FSI-GTL framework combines signal processing, graph representation learning, and Transformer-based attention mechanisms in a single end-to-end diagnosis framework. The basic principle of this framework is that fault occurrence causes distinct energy distributions across multiple frequency bands, and there are correlations between them in a spectral manner that is helpful to diagnosis. Instead of treating the transformed signals as images, the proposed method represents the spectral information as a graph where spectral interactions and sources are modeled simultaneously through a self-attention mechanism.

The total architecture consists of four levels of learning stages. The first stage transforms the time-domain vibration signal (collected from CWRU bearing data) into time–frequency-locality images through a group of combination spectral tools [Short Time Fourier Transform, Wavelet packet transformation, Empirical Mode Decomposition (EMD), and Cepstral analysis]. The techniques can enhance spectral bands that strongly relate to bearing faults, which are not easy to identify in the time-domain waveform. Simultaneously considering multiple time-frequency-locality representations makes it easy to analyze distributed as well as local frequency information in cross-bearing faults.

Second, discriminative frequency-domain features are computed from the image of the signal generated and used for constructing the graph representations. In the proposed graph construction paradigm, salient frequency regions each correspond to a node, and an edge represents a similarity relation among spectral regions between two nodes. Such graph representations clearly learn structure relations among various fault specific frequency components rather than meaningless image pixels, allowing the topological knowledge about fault development being preserved.

Third, the GNN is adopted to learn local structure relations according to the constructed graph. Information is propagated within connected spectral regions by iterative neighborhood aggregation in the GNN, thereby learning topology-sensitive node embedding. Step 3 allows the framework to capture the complicated relationship between adjacent frequency components and learn a reliable feature of spectral structure driven by fault.

Fourth, the graph embeddings generated by GNN are passed into a Transformer encoder. Graph convolution can learn local structural information well, but it lacks the long-range capacity for long-distance dependency. The Transformer encoder effectively uses multi-head self-attention to generate the long-distance dependencies over the entire graph representation, so that the entire framework can find global fault signatures located across various frequency domains and operating conditions.

Finally, combining the graph-based structure feature with the Transformer-based context feature gives the complete fault representation. This fused feature vector contains the local topological information extracted by GNN and global context information extracted by Transformer. A fully-connected classifier is employed to classify bearing health status, including normal, inner-race fault, outer-race fault, and ball fault states.

The proposed FSI-GTL framework addresses the major limitations of previous research as mentioned above and surveyed in Section 2. Different from former CNN-based methods, the proposed FSI-GTL framework explicitly captures the structural dependencies among multiple frequency regions by graph learning; Different from ordinary GNN-based approaches, the FSI-GTL framework makes use of a Transformer-based self-attention mechanism to adaptively capture the long-range relationships between frequency sub-bands; Furthermore, the integration of multiple frequency-aware signal imaging techniques is adopted to further improve fault discriminability and robustness.

The workflow of the proposed FSI-GTL framework can be mathematically expressed as


$$\begin{array}{l} Y=C\left(FT\left(FG\left(G\left(FE\left(I\left(X\right)\right)\right)\right)\right)\right) \end{array}$$

where

- *X* denotes the preprocessed vibration signal,
- *I*(·) denotes frequency-aware signal image generation,
- *FE*(·) denotes frequency feature extraction,
- *G*(·) denotes graph construction,
- *FG*(·) denotes graph neural network learning,
- *FT*(·) denotes Transformer-based global feature learning,
- *C*(·) denotes fault classification,
- *Y* represents the predicted bearing condition.

Thus, the proposed FSI-GTL framework establishes a hierarchical learning pipeline that progressively transforms raw vibration measurements into discriminative fault representations by jointly exploiting frequency information, graph topology, and attention-driven contextual relationships.

### 3.2 Data acquisition and signal preprocessing

The well-known Case Western Reserve University (CWRU) bearing fault dataset, one of the most popular benchmark datasets in intelligent fault diagnosis research, is used to perform an experimental analysis. It contains vibration measurements acquired on bearings with different fault severity and working conditions, including bearings with inner-race fault, outer-race fault, and rolling element defect.

The acquired vibration signal is first segmented into fixed-length windows:


$$\begin{array}{l} Si=\left\{x_{i},x_{i+1},\ldots ,x_{i+L-1}\right\} \end{array}$$

where L denotes the segment length.

To eliminate measurement inconsistencies and improve feature stability, z-score normalization is applied:


$$\begin{array}{l} X_{norm}=\frac{X-\mu \;}{\sigma } \end{array}$$

The noise was then filtered using a Butterworth band-pass filter so that only the fault characteristic frequencies within the signal were extracted. The preprocessed vibration signal is denoted as


$$\begin{array}{l} X_{p} \end{array}$$

and serves as the input for frequency-aware signal transformation.

### 3.3 Frequency-aware signal-to-image transformation

Typical bearing faults are a part of localized energy variation in a common frequency domain. Thus, the original vibration signal may lack enough distinctive fault-related information that is necessary for accurate diagnosis. To get around this drawback, the proposed framework converts the vibration signal to frequency-based images using different spectral decompositions.

#### 3.3.1 Short-time Fourier transform representation

The short-time Fourier transform (STFT) generates localized time-frequency information:


$$\begin{array}{l} STFT\left(\tau ,f\right)=\int_{-\infty }^{\infty }X_{p}\left(t\right)w\left(t-\tau \right)e^{-j2\pi ft}dt \end{array}$$

where w(t) denotes the sliding window function.

The corresponding spectrogram image is obtained as


$$\begin{array}{l} I_{STFT}=|STFT\left(\tau ,f\right){|}^{2} \end{array}$$

which reveals fault-related energy distributions across time and frequency domains.

#### 3.3.2 Wavelet packet transform representation

To capture multi-resolution frequency characteristics, WPT decomposes the vibration signal into multiple sub-bands:


$$\begin{array}{l} W_{j,k}=\int X_{p}\left(t\right)\psi_{j,k}\left(t\right)dt \end{array}$$

where j and k represent the decomposition level and node index, respectively.

The energy distribution of each frequency band is computed as


$$\begin{array}{l} E_{k}=\sum |W_{j,k}{|}^{2} \end{array}$$

and converted into a visual energy map.

#### 3.3.3 Empirical mode decomposition representation

Empirical mode decomposition adaptively separates the vibration signal into intrinsic oscillatory modes:


$$\begin{array}{l} X_{p}\left(t\right)=\overset{m}{\underset{i=1}{\sum }}\;IMF_{i}\left(t\right)+r\left(t\right) \end{array}$$

where *IMF*_*i*_ represents intrinsic mode functions and r(t) denotes the residual signal.

The energy distribution of the generated IMFs is transformed into an image representation.

#### 3.3.4 Cepstral representation

Cepstral analysis is employed to identify periodic fault impulses hidden within vibration spectra:


$$\begin{array}{l} C\left(q\right)=F^{-1}\left[log\left(|F\left(X_{p}\right)|\right)\right] \end{array}$$

where:

- *X*_*p*_ denotes the preprocessed vibration signal,
- *F*(·) denotes the Fourier transform,
- *F*^−1^(·) denotes the inverse Fourier transform,
- *q* represents the quefrency, which corresponds to the time delay associated with periodic spectral components.

The generated cepstral image highlights repetitive fault signatures associated with bearing degradation.

The final frequency-aware image representation is obtained through the fusion of STFT, WPT, EMD, and cepstral images:


$$\begin{array}{l} I_{f}=I_{STFT}⊕I_{WPT}⊕I_{EMD}⊕I_{CEP} \end{array}$$

where ⊕ denotes the multi-representation image fusion operation described in Section 3.3.5.

#### 3.3.5 Multi-representation image fusion

Although each type of signal transformation can detect some specific fault characteristics, no time-frequency representation can extract all types of spectral characteristics for the failure mode of rolling bearing. The STFT can perceive local time-frequency features but is subject to fixed time-frequency resolution. WPT provides high multi-resolution frequency resolution and good frequency localization; EEMD can extract the inherent time series modes adaptively and automatically from the non-stationary vibration signal, and Cepstrum analysis enhances prominent periodicity of gear fault impulses and harmonic components. Therefore, these time-frequency distributions yield varying aspects of failure details.

To address this constraint, the developed FSI-GTL also designs a multi-representation image fusion (MRIF) algorithm for efficiently fusing the beneficial information from different spectral images (i.e., the STFT, WPT, EMD, and cepstral spectral) into the fused frequency-aware image. It is intended to retain all useful and defect-sensitive information by considering the characteristics of the spectral representations.

Let the four generated signal-image representations be denoted as


$$\begin{array}{l} I_{STFT},I_{WPT},I_{EMD},I_{CEP} \end{array}$$

where:

- *I*_*STFT*_ represents the spectrogram generated by STFT,
- *I*_*WPT*_ represents the wavelet packet energy map,
- *I*_*EMD*_ represents the IMF-based energy image,
- *I*_*CEP*_ represents the cepstral image.

Since the intensity ranges and frequency distributions of these representations vary significantly, direct fusion may introduce representation bias. Therefore, each image is first normalized independently using min-max normalization:


$$\begin{array}{l} I_{k}^{norm}=\frac{I_{k}-I_{k}^{\min}}{I_{k}^{\max}-I_{k}^{\min}} \end{array}$$

where:

- $I_{k}^{norm}$ denotes the normalized image,
- $I_{k}^{\min}$ and $I_{k}^{\max}$ represent the minimum and maximum pixel values,
- *k*∈{*STFT, WPT, EMD, CEP*}

The normalization process scales all image intensities into the range [0, 1], ensuring balanced contributions during the fusion stage.

After normalization, all representations are resized to a common spatial dimension of M × N pixels to maintain dimensional consistency:


$$\begin{array}{l} I_{k}^{resize}=Resize\left(I_{k}^{norm},M,N\right) \end{array}$$

to ensure dimensional consistency during fusion.

##### 3.3.5.1 Adaptive attention-based image fusion

Unlike conventional image fusion methods that assign fixed weights to each representation, the proposed framework employs an adaptive attention mechanism to automatically determine the contribution of each transformed image. This enables the network to emphasize the most informative frequency representation according to the operating condition and fault characteristics.

For each transformed image, a compact global representation is first obtained using global average pooling (GAP):


$$\begin{array}{l} z_{k}=GAP\left(I_{k}^{resize}\right) \end{array}$$

The pooled features are passed through a lightweight attention network to compute attention scores:


$$\begin{array}{l} sk=W_{2}\delta \left(W_{1}z_{k}\right) \end{array}$$

where

- *W*_1_ and *W*_2_ are learnable weight matrices,
- δ(·) denotes the ReLU activation function.

The attention scores are normalized using the Softmax function to obtain adaptive fusion weights:


$$\begin{array}{l} \alpha_{k}=\frac{exp\left(s_{k}\right)}{\sum_{j=1}^{4}exp\left(S_{j}\right)} \end{array}$$

subject to


$$\begin{array}{l} \overset{4}{\underset{k=1}{\sum }}\alpha_{k},\;\alpha_{k}=1,\;\alpha_{k}\geq 0 \end{array}$$

The fused frequency-aware image is then computed as


$$\begin{array}{l} I_{F}=\alpha_{STFT}I_{STFT}^{resize}+\alpha_{WPT}I_{WPT}^{resize}+\alpha_{EMD}I_{EMD}^{resize}+\alpha_{CEP}I_{CEP}^{resize}. \end{array}$$

Unlike fixed-weight fusion, the proposed adaptive strategy dynamically learns the relative importance of each spectral representation during network training. Consequently, the framework can automatically emphasize the most discriminative transformation while suppressing less informative representations, thereby improving robustness under varying operating conditions and noise levels.

To further enhance fault-sensitive structures, histogram equalization is applied:


$$\begin{array}{l} I_{Final}=HE\left(I_{F}\right) \end{array}$$

where HE(·) denotes the histogram equalization operation.

The resulting image *I*_*Final*_ contains complementary time-frequency information aggregated from multiple signal representations and serves as the input for the subsequent graph construction and feature learning stages. By adaptively integrating localized spectral patterns, multiresolution frequency characteristics, intrinsic oscillatory modes, and periodic fault signatures, the proposed MRIF module generates a more discriminative and robust frequency-aware representation for the GNN and Transformer encoder.

### 3.4 Frequency feature extraction

The fused frequency-aware image contains rich spectral information describing bearing operating conditions. To construct graph representations, discriminative frequency-domain features are extracted from significant spectral regions.

The extracted feature vector is represented as


$$\begin{array}{l} F=\left\{f_{1},f_{2},\ldots ,f_{d}\right\} \end{array}$$

where d denotes the feature dimension.

#### 3.4.1 Spectral energy

Spectral energy is defined as the sum of the power of the whole signal distribution over the frequency range. As far as fault in bearing is concerned, which is characterized by the additional impacts of vibration, the masses of energy over some frequency bands increase. Therefore, spectral energy is also a useful indicator response variable for the degree of fault severity and abnormality detection.

The spectral energy is calculated as


$$\begin{array}{l} E=\overset{N}{\underset{i=1}{\sum }}|X_{i}{|}^{2} \end{array}$$

where:

- *X*_*i*_ denotes the spectral amplitude at the *i*th frequency bin,
- N denotes the total number of frequency bins.

A good bearing typically produces approximately consistent energy level, while the fault bearing producing much higher energy by the impacts of the fault. Therefore, the energy spectrum can differentiate the normal operating state and faulty condition.

#### 3.4.2 Spectral entropy

Spectral entropy is a measure of how disorder or complexity of the frequency spectrum is. Bearing degradation modifies vibration in an unpredictable manner, pushing energy into many frequencies and increasing spectral uncertainty.

The probability distribution of spectral components is computed as


$$\begin{array}{l} p_{i}=\frac{|X_{i}|}{\sum_{j=1}^{N}|X_{j}|} \end{array}$$

The spectral entropy is then calculated as


$$H=-\sum_{i=1}^{N}p_{i}log(p_{i})$$

where:

- *p*_*i*_ represents the normalized spectral probability.

A smaller entropy value corresponds to a “sharper” distribution of energy within the spectrum, whereas higher values suggest more complex and “disordered” spectral data. Given that bearing failures are expected to result in a more randomly distributed spectrum, entropy values can serve as important “hints” as to the state and failure of the system.

#### 3.4.3 Spectral kurtosis

The spectral kurtosis is a measure of vibration signal impulsiveness, which can be used for detection of local bearing defects. Local mechanical defects manifest themselves as transient shock impulses creating “spikes” on a vibration signal frequency spectrum.

Spectral kurtosis is computed as


$$\begin{array}{l} SK=\frac{1}{N}\overset{N}{\underset{i=1}{\sum }}{\left(\frac{X_{i}-\mu }{\sigma }\right)}^{4} \end{array}$$

Where

- μ denotes the spectral mean,
- σ denotes the spectral standard deviation.

Impulsive fault signatures typically have a higher kurtosis value; healthy bearings are relatively smooth and thus higher averages of kurtosis statistics are expected. Hence, spectral kurtosis is very sensitive for early fault detection.

#### 3.4.4 Spectral centroid

The spectral centroid specifies the center of mass of the magnitude of the frequency spectrum. It indicates where the main frequency component is placed in the spectrum. The faults of bearing usually result in moving spectrum energy to the high-frequency side because of the repeated impact and the resonance phenomenon.

The spectral centroid is calculated as


$$\begin{array}{l} SC=\frac{\sum_{i=1}^{N}f_{i}|X_{i}|}{\sum_{i=1}^{N}|X_{i}|} \end{array}$$

Where

- *f*_*i*_ denotes the frequency corresponding to the *i*th spectral component.

A shift in the spectral centroid indicates changes in vibration characteristics caused by bearing degradation. Therefore, the spectral centroid provides useful information regarding the frequency localization of fault-induced energy concentrations.

#### 3.4.5 Band energy ratio

The energy ratio on these bands indicates the amount of energy in fault sensitive frequency region. It is usually within a range of relative energy corresponding to characteristic frequencies of inner race, outer race, and rolling elements.

Let *B*_*k*_ denote the *k*th frequency band. The energy within the band is calculated as


$$\begin{array}{l} E_{k}=\underset{f_{i}\in B_{k}}{\sum }|X_{i}{|}^{2} \end{array}$$

The band energy ratio is then computed as


$$\begin{array}{l} BER_{k}=\frac{E_{k}}{\sum_{j=1}^{M}E_{j}} \end{array}$$

Where

- M denotes the total number of frequency bands.

The parameter can accurately locate the frequency distribution in fault mode. It can distinguish faults of different types well. Since an inside run race defect will have its energy distributed to particular frequency bands, it will show up in the spectral centroid.

#### 3.4.6 Spectral spread

Observe that the spectral centroid measures where the main frequencies are centered. However, it does not indicate how distributed the energies are around its middle. The spectral spread tries to quantify the width of the spectral distribution, and therefore provides information about the frequency range of the fault's signatures.

The spectral spread is calculated as


$$\begin{array}{l} SS=\sqrt{\frac{\sum_{i=1}^{N}{\left(f_{i}-SC\right)}^{2}|X_{i}|}{\sum_{i=1}^{N}|X_{i}|}} \end{array}$$

Where

- SC denotes the spectral centroid.

The spectrum of a healthy bearing has a narrow distribution of frequencies, and a measurement of a bearing fault will display the spread of frequencies due to harmonic components, resonance, etc. This spectral spread, therefore, provides a test of the spread of frequencies arising due to a bearing fault.

##### 3.4.6.1 Feature vector formation

The extracted frequency descriptors are combined to form the final feature vector:


$$\begin{array}{l} F=\left[E,\;H,\;SK,\;SC,\;BER,SS\right] \end{array}$$

Where

- *E* = spectral energy,
- *H* = spectral entropy,
- SK = spectral kurtosis,
- SC = spectral centroid,
- BER = band energy ratio,
- SS = spectral spread.

All above features are describing the amplitude, complexity, impulsivity, concentration and distribution attributes of vibration signal. It could be observed that, they are representing different aspects of bearing behavior. So, information overlap is not that big among those features. The concatenated features vector will be a powerful representative for the purpose of graph generation and GNN learning. The concatenated feature vector is essentially a regulated and more compact feature space to preserve good fault-separability within the frequency aware image to the later stage analysis to speed up.

The frequency-domain descriptors extracted in this stage are utilized to analyze the discriminative characteristics of the transformed vibration signals and to facilitate adaptive image fusion. These descriptors are not directly assigned as graph node attributes. Instead, after frequency-aware image generation and patch partitioning, graph node features are extracted from the corresponding two-dimensional image regions, as described in Section 3.5.1.

### 3.5 Graph construction strategy

Unlike conventional image-based approaches, the proposed framework explicitly models structural relationships among frequency regions using graph representations.

A graph is defined as


$$\begin{array}{l} G=\left(V,E,A\right) \end{array}$$

where V represents nodes, E denotes edges, and A is the adjacency matrix.

Each significant frequency region extracted from the fused image is treated as a graph node:


$$\begin{array}{l} V=\left\{v_{1},v_{2},\ldots ,v_{n}\right\} \end{array}$$

#### 3.5.1 Node generation from frequency-aware images

The frequency-preserved image obtained after multi-representation image fusion is a spectral spatial image that has abundant spectral information in distinct spatial locations. To avoid over-computing cost caused by using every pixel as a node in the graph, we propose dividing the fused spectral image into multiple nonoverlapping spectral regions.

Let the fused image be represented as


$$\begin{array}{l} I_{F}\in R^{M\times N} \end{array}$$

Where

- M denotes image height,
- N denotes image width.

The image is divided into m × n spectral patches:


$$\begin{array}{l} I_{F}=\left\{ROI_{1},ROI_{2},\ldots ,ROI_{K}\right\} \end{array}$$

Where


$$\begin{array}{l} K=m\times n \end{array}$$

and *ROI*_*K*_ represents the *k*^th^ region of interest.

Each spectral region corresponds to a graph node:


$$\begin{array}{l} v_{k}=\left\{ROI_{k}\right\} \end{array}$$

Thus, the node set becomes


$$\begin{array}{l} V=\left\{v_{1},v_{2},\ldots ,v_{K}\right\} \end{array}$$

This patch-based node generation strategy offers several advantages:

- Reduces graph complexity compared with pixel-level graphs.
- Preserves local spectral structures.
- Maintains frequency-band relationships.
- Improves computational efficiency.

For each spectral patch, a low-dimensional image descriptor is extracted to characterize the local structural and textural information contained within the corresponding frequency region. Unlike the frequency-domain descriptors presented in Section 3.4, which are computed directly from one-dimensional transformed vibration signals, the graph node attributes are derived from two-dimensional image patches to preserve mathematical consistency during graph construction.

For the *k*th spectral patch, the node feature vector is defined as


$$\begin{array}{l} h_{k}=\left[\mu_{k},\sigma_{k},G_{k},LBP_{k}\right] \end{array}$$

Where

- μ_*k*_ denotes the mean pixel intensity,
- σ_*k*_ denotes the standard deviation,
- *G*_*k*_ denotes the average gradient magnitude,
- *LBP*_*k*_ represents the local binary pattern texture descriptor.

The mean intensity of each image patch is computed as


$$\begin{array}{l} \mu_{k}=\frac{1}{MN}\overset{M}{\underset{i=1}{\sum }}\overset{N}{\underset{j=1}{\sum }}I\left(i,j\right) \end{array}$$

The standard deviation is computed as


$$\begin{array}{l} \sigma_{k}=\sqrt{\frac{1}{MN}{\overset{M}{\underset{i=1}{\sum }}\overset{N}{\underset{j=1}{\sum }}\left(I\left(i,j\right)-\mu_{k}\right)}^{2}} \end{array}$$

The average gradient magnitude is


$$\begin{array}{l} G_{k}=\frac{1}{MN}\overset{M}{\underset{i=1}{\sum }}\overset{N}{\underset{j=1}{\sum }}G_{x}{\left(i,j\right)}^{2}+G_{y}{\left(i,j\right)}^{2} \end{array}$$

Where *G*_*x*_ and *G*_*y*_ denote the horizontal and vertical image gradients obtained using the Sobel operator.

The local binary pattern (LBP) descriptor is extracted from each image patch to encode local texture variations associated with fault-induced frequency patterns. The concatenation of these descriptors forms the final node representation.


$$\begin{array}{l} H={\left[h_{1},h_{2},\ldots ,h_{K}\right]}^{T} \end{array}$$

Where


$$\begin{array}{l} H\in R^{K\times d} \end{array}$$

and *d* denotes the dimensionality of the image feature vector.

This patch-level representation preserves local spatial structures and texture characteristics while significantly reducing graph complexity compared with pixel-level graph construction.

Based on preliminary sensitivity analysis (Section 5.12), the neighborhood size was empirically set to k = 8, which provided the best trade-off between graph connectivity and diagnostic accuracy.

#### 3.5.2 Edge generation using adaptive k-nearest neighbor graph

When graph nodes have been generated, spectral region interdependencies are set. Bearing failure could result in cross-correlated spectral responses across several spectral bands. Edges connect consecutive spectral regions with similar spectral characteristics.

To identify these relationships, Euclidean distance is first computed between node feature vectors:


$$\begin{array}{l} D_{ij}=∥h_{i}-h_{j}{∥}^{2} \end{array}$$

Where

- *h*_*i*_ and *h*_*j*_ denote node feature vectors.

For each node *v*_*i*_, the k most similar neighboring nodes are identified using the k-nearest neighbor (k-NN) strategy.

The edge connectivity rule is defined as


$$E_{ij}=\{\begin{array}{c} 1,\;v_{j}\in KNN(v_{i})\\ 0,\;otherwise\; \end{array}$$

Where

- KNN(*v*_*i*_) denotes the set of k nearest neighbors of node *v*_*i*_.

The k-NN graph construction prevents excessive graph density and ensures that only meaningful relationships are preserved.

After neighborhood identification, Gaussian similarity weighting is employed to quantify the strength of each connection:


$$\begin{array}{l} A_{ij}=exp\left(\frac{∥h_{i}-h_{j}{∥}^{2}}{2\sigma^{2}}\right) \end{array}$$

Where σ controls neighborhood connectivity.

- *A*_*ij*_ denotes edge weight,
- σ controls similarity sensitivity.

The weighted adjacency matrix becomes


$$\begin{array}{l} A={\left[A_{ij}\right]}_{K\times K} \end{array}$$

The Gaussian weighting mechanism provides several advantages:

1. Strongly connected nodes correspond to highly similar spectral regions.
2. Weakly related frequency regions receive lower connectivity weights.
3. Noise-induced relationships are naturally suppressed.
4. Graph topology becomes more robust under varying operating conditions.

To improve graph stability, self-loops are introduced:


$$\begin{array}{l} Â+A+I \end{array}$$

Where *I* denotes the identity matrix.

The corresponding degree matrix is computed as


$$\begin{array}{l} D_{ij}=\underset{j}{\sum }{Â}_{ij} \end{array}$$

which is later utilized during graph convolution operations.

The Gaussian bandwidth parameter was set to σ = 1.0 according to the sensitivity analysis presented in Section 5.12.

#### 3.5.3 Graph representation of frequency relationships

This resultant graph representation forms a spectral structural representation capturing the local and global spectral information under bearing fault conditions. Unlike image-based approaches, which consider only local frequency features individually, the proposed variant graph formulation takes the structural connectivity between different frequency components into account.

The final graph can therefore be expressed as


$$\begin{array}{l} G=\left(V,E,A\right) \end{array}$$

with


$$\begin{array}{lll} V & = & \left\{v_{1},v_{2},\ldots ,v_{K}\right\}\\ E & = & \left\{\left(v_{i},v_{j}\right)\right\}\\ A & = & \left[A_{ij}\right] \end{array}$$

where node features encode local image appearance and texture characteristics extracted from frequency-aware image patches, while edge weights describe similarity relationships between neighboring spectral regions.

Consequently, the graph constructed according to the suggested method would contain significantly more numerous and rich fault-associated information than image schemes. By inheriting local spectral information as well as topological correlations between neighboring spectral bands, this graph could provide a more informative input to the feature learning step with a GNN. This input would become part of the proposed FSI-GTL framework to utilize the multi-scale interpretation of intricate faults presented in various spectral bands.

### 3.6 Graph neural network feature learning

The constructed graph is processed using GNNs to learn topological relationships among spectral regions.

For each graph convolution layer, node representations are updated according to


$$\begin{array}{l} H^{\left(l+1\right)}=\sigma \left({\hat{D}}^{-\frac{1}{2}}\;{Â\hat{D}}^{-\frac{1}{2}}H^{\left(l\right)}W^{\left(l\right)}\right) \end{array}$$

Where $Â=A+I,\;\hat{D}$ denotes the degree matrix, and *W*^(*l*)^ represents trainable parameters.

The graph embedding generated by the GNN is expressed as


$$\begin{array}{l} Z_{G}=GNN\left(G\right) \end{array}$$

which captures local structural information associated with bearing fault evolution.

### 3.7 Transformer encoder for global dependency learning

Concretely, while the GNN can represent well the local structural dependencies of neighboring frequency components, it only carries out the information propagation among the local graph neighbors. It is well known that in rolling bearing fault diagnosis, long-term spectral components are always loaded with important fault information, and long-range interaction among spectral components is always rich in diagnostic significance. Hence, the utilization of only graph convolution will be insufficient for the representation of long-range fault dependencies. Based on this, we add a Transformer encoder after the graph feature learning module in the proposed FSI-GTL framework to carry out modeling of the overall graph-node relationships, and aggregate global contextual information of the whole spectra through the self-attention mechanism.

Let the graph embedding generated by the GNN be represented as


$$\begin{array}{l} Z_{G}\in R^{K\times d} \end{array}$$

Where K denotes the number of graph nodes, and d denotes the embedding dimension.

The transformer encoder consists of three major components: positional encoding, multi-head self-attention, and feed-forward learning.

#### 3.7.1 Positional encoding

Before self-attention, each graph node embedding is augmented with a two-dimensional spatial positional encoding derived from the corresponding patch location in the fused frequency-aware image. Since graph nodes originate from fixed image patches rather than an arbitrary sequence, each node is assigned a unique spatial coordinate. (*x*_*i*_, *y*_*i*_), representing its row and column position within the image grid. The positional representation preserves the spatial arrangement of frequency regions and enables the Transformer to distinguish neighboring and distant spectral locations.

The positional embedding of node iii is expressed as


$$\begin{array}{l} z_{i}=h_{i}+p_{i} \end{array}$$

Where

- *h*_*i*_ denotes the graph node embedding,
- *p*_*i*_ denotes the learnable two-dimensional positional embedding.

For a patch located at coordinate (*x*_*i*_, *y*_*i*_), the positional embedding is defined as


$$\begin{array}{l} p_{i}=P_{x}\left(x_{i}\right)+P_{y}\left(y_{i}\right) \end{array}$$

Where

- *P*_*x*_(.) represents the row embedding,
- *P*_*y*_(.) represents the column embedding.

The resulting node representation


$$\begin{array}{l} Z=\left\{z_{1},z_{2},\ldots ,z_{K}\right\} \end{array}$$

is subsequently processed by the Transformer encoder using multi-head self-attention.

Unlike conventional sequence-based positional encoding, the proposed two-dimensional spatial positional embedding preserves the geometric arrangement of frequency regions extracted from the fused spectral image, thereby providing a more meaningful positional representation for graph-based fault diagnosis.

#### 3.7.2 Multi-head self-attention

The core learning mechanism of the Transformer encoder is multi-head self-attention. Unlike graph convolution, which only aggregates information from neighboring nodes, self-attention allows each node to interact with every other node in the graph.

For the input representation *Z*_0_, query, key, and value matrices are generated as


$$\begin{array}{lll} Q & = & Z_{G}W_{Q}\\ K & = & Z_{G}W_{K}\\ V & = & Z_{G}W_{V} \end{array}$$

The self-attention operation is defined as


$$\begin{array}{l} Attention\left(Q,K,V\right)=Softmax\left(\frac{QK^{T}}{\sqrt{d_{k}}}\right)V \end{array}$$

Where *d*_*k*_ denotes the key dimension.

Much attention mechanism has emphasized the spectrum range relevant to the fault feature more. Therefore, spectrum coupling is given more consideration in feature extraction.

To capture diverse relationships, multiple attention heads are employed. The output of the *h*th attention head is


$$\begin{array}{l} head_{h}=Attention\left(Q_{h},K_{h},V_{h}\right) \end{array}$$

The outputs of all attention heads are concatenated and projected:


$$\begin{array}{l} MHA=Concat\left(head_{1},head_{2},\ldots ,head_{H}\right)W_{O} \end{array}$$

Where

- *H* denotes the number of attention heads,
- *W*_*O*_ denotes the output projection matrix.

Through multi-head attention, the transformer simultaneously learns different types of global frequency relationships and fault interactions.

#### 3.7.3 Feed forward network

After self-attention learning, the resulting representation is passed through a position-wise feed-forward network to enhance nonlinear feature extraction capability.

The feed-forward transformation is expressed as


$$\begin{array}{l} FFN\left(x\right)=W_{2}ReLU\left(W_{1}x+b_{1}\right)+b_{2} \end{array}$$

Where

- *W*_1_ and *W*_2_ denote trainable weight matrices,
- *b*_1_ and *b*_2_ denote bias vectors.

Residual connections and layer normalization are incorporated to improve training stability:


$$\begin{array}{lll} Z_{1} & = & LayerNorm\left(Z_{0}+MHA\right)\\ Z_{T} & = & LayerNorm\left(Z_{1}+FFN\left(Z_{1}\right)\right) \end{array}$$

Where *Z*_*T*_ denotes the final transformer representation.

Therefore, the transformer encoder encodes the long-range contextual relationship among frequency regions and then builds the globally aware fault characteristics, which complement the local structural information captured by the GNN.

### 3.8 Feature fusion and fault classification

The final fault representation is obtained by combining graph-derived structural features and Transformer-derived contextual features:


$$\begin{array}{l} Z_{F}=Z_{G}⊕Z_{T} \end{array}$$

The fused feature vector is subsequently passed through fully connected layers and a Softmax classifier:


$$\begin{array}{l} P\left(y|X\right)=Softmax\left(W_{f}Z_{F}+b_{f}\right) \end{array}$$

Where *W*_*f*_ and *b*_*f*_ denote classifier parameters.

The predicted fault class is determined as


$$\begin{array}{l} ŷ=\arg maxP\left(y|X\right) \end{array}$$

The framework classifies bearing conditions into four categories:

- Normal condition.
- Inner-race fault.
- Outer-race fault.
- Ball defect fault.

The network parameters are optimized using the cross-entropy loss function:


$$\begin{array}{l} L=-\frac{1}{N}\;\overset{N}{\underset{i=1}{\sum }}\overset{C}{\underset{c=1}{\sum }}y_{ic}log\left({ŷ}_{ic}\right) \end{array}$$

Where *C* denotes the number of bearing health states.

Using such integrated framework, the FSI-GTL could jointly use frequency-aware visual features, graph-based structural relations and transformer-based global dependence to improve its power of fault discrimination and diagnostic ability under varying working conditions in a uniform manner. The complete procedure of the proposed FSI-GTL framework is summarized in Algorithm 1.

Algorithm 1 Frequency-aware signal image representation and graph transformer learning (FSI-GTL)

  Input: Preprocessed vibration signal *X*_*p*_
  Output: Predicted fault class ŷ
 1:  Acquire vibration signals from the CWRU dataset.
 2:  Perform normalization and Butterworth filtering.
 3:  Generate STFT spectrogram image *I*_STFT_.
 4:  Generate WPT energy image *I*_WPT_.
 5:  Generate EMD-based IMF image *I*_EMD_.
 6:  Generate cepstral image *I*_CEP_.
 7:  Normalize and resize all image representations.
 8:  Fuse transformed images to obtain frequency-aware image *I*_*F*_.
 9:  Extract spectral energy, entropy, kurtosis, spectral centroid, band energy ratio, and spectral spread features.
 10:  Partition the fused image into spectral patches.
 11:  Generate graph nodes from image regions.
 12:  Construct an adaptive *k*-nearest-neighbor graph.
 13:  Compute a Gaussian-weighted adjacency matrix.
 14:  Apply Graph Neural Network learning.
 15:  Generate graph embedding *Z*_*G*_.
 16:  Apply positional encoding.
 17:  Perform multi-head self-attention.
 18:  Apply a feed-forward network.
 19:  Generate transformer embedding *Z*_*T*_.
 20:  Perform adaptive feature fusion.
 21:  Apply a fully connected classification layer.
 22:  Compute Softmax probabilities.
 23:  Predict bearing fault category.
 24:  Update network parameters using Adam optimization.
 25:  Repeat until convergence.
  Return: Predicted fault class ŷ

### 3.9 Algorithm of proposed FSI-GTL framework

### 3.10 Computational complexity analysis

The computational complexity of the proposed FSI-GTL framework is analyzed to evaluate its scalability and suitability for industrial deployment.

#### 3.10.1 Frequency-aware signal transformation

The dominant complexity associated with STFT, WPT, EMD, and cepstral analysis is approximated as


$$\begin{array}{l} O\left(NlogN\right) \end{array}$$

where *N* denotes the vibration signal length.

#### 3.10.2 Graph construction

For K graph nodes, pairwise similarity computation requires


$$\begin{array}{l} O\left(K^{2}\right) \end{array}$$

operations.

The k-nearest neighbor search requires


$$\begin{array}{l} O\left(KlogK\right) \end{array}$$

Therefore, graph construction complexity is approximated as


$$\begin{array}{l} O\left(K^{2}\right) \end{array}$$

#### 3.10.3 Graph neural network learning

For a graph containing |E|| edges and feature dimension d, graph convolution requires


$$\begin{array}{l} O\left(|E|d\right) \end{array}$$

per layer.

For LGL_GLG graph convolution layers, the total complexity becomes


$$\begin{array}{l} O\left(L_{G}|E|d\right) \end{array}$$

#### 3.10.4 Transformer encoder

The Transformer self-attention mechanism computes pairwise interactions among all graph nodes.

For *K* nodes and embedding dimension d


$$\begin{array}{l} O\left(K^{2}d\right) \end{array}$$

operations are required.

For *L*_*T*_ Transformer layers, the complexity becomes


$$\begin{array}{l} O\left(L_{T}K^{2}d\right) \end{array}$$

#### 3.10.5 Adaptive feature fusion

The adaptive fusion and classification stages require


$$\begin{array}{l} O\left(K_{d}\right) \end{array}$$

which is negligible compared with graph construction and self-attention operations.

#### 3.10.6 Overall complexity

Combining all major components, the total complexity of the proposed framework is


$$\begin{array}{l} O\left(NlogN\right)+O\left(K^{2}\right)+O\left(L_{G}|E|d\right)+O\left(L_{T}K^{2}d\right) \end{array}$$

Since transformer self-attention and graph similarity computation dominate the processing cost, the overall complexity can be approximated as


$$\begin{array}{l} O\left(K^{2}d\right) \end{array}$$

The practical implementation where an image patch is used instead of pixels to construct the graph node can downsize the graph significantly and then alleviate the computational burden of graph processing. Hence, the proposed FSI-GTL framework can achieve a balanced trade-off between computational efficiency and diagnostic performance by fully utilizing the benefits of frequency aware signal imaging, graph learning and Transformer-based global dependency modeling.

## 4 Experimental setup

### 4.1 Experimental environment

The proposed FSI-GTL framework was implemented in Python using the PyTorch framework. Graph-based operations were performed using the PyTorch Geometric library. Transformer-based feature learning was implemented using standard deep learning building blocks. All experiments were run on a machine with an Intel Core I9 processor, 64 GB RAM, and an NVIDIA GTX- series GPU. GPU acceleration was used to train the graph learning and the Transformer modules.

The entire experimental pipeline includes four consecutive steps: frequency-aware signal image generation, graph construction, GNN learning, and Transformer-based global dependency learning. The Adam optimizer was applied in training due to its good adaptation to learning and stable convergence. Early stopping and dropout regularization were used to avoid overfitting.

The overall experimental workflow can be represented as


$$\begin{array}{l} X\to I_{F}\to Z_{G}\to Z_{T}\to Z_{F}\to ŷ \end{array}$$

Where

- *X* denotes the preprocessed vibration signal,
- *I*_*F*_ denotes the fused frequency-aware image,
- G denotes the graph representation,
- *Z*_*G*_ denotes graph embeddings,
- *Z*_*T*_ denotes Transformer embeddings,
- *Z*_*F*_ denotes fused features,
- ŷ denotes the predicted fault class.

This experimental configuration enables systematic evaluation of each component of the proposed FSI-GTL framework.

### 4.2 Dataset description

The data for this experiment are from the benchmark bearing fault data collected by the Bearing Data Center of CWRU. The CWRU dataset is selected as the benchmark dataset of intelligent fault diagnosis because it has been popularly regarded as a benchmark dataset in the current status of state of art diagnosis studies and the vibration data is available for a wide range of fault types, fault severity, rotating speeds and operational states.

The experimental test rig employed was fitted with an induction motor (2hp), a torque transducer, a dynamometer, and deep-groove ball bearings. Faults were introduced by EDM to artificially simulate various degrees of bearing damage. Vibration data was gathered from acceleration sensors fitted to the drive end and fan end of the rig.

Let the acquired vibration signal be represented as


$$\begin{array}{l} X\left(t\right)=\left\{x_{1},x_{2},\ldots ,x_{N}\right\} \end{array}$$

where *N* denotes the total number of vibration samples.

The dataset contains four primary bearing health conditions:

Class DescriptionNormal Healthy bearingIRF Inner-race faultORF Outer-race faultBF Ball defect fault

The vibration signals were recorded using two sampling frequencies:


$$\begin{array}{l} f_{s}=12\;kHz \end{array}$$

and


$$\begin{array}{l} fs=48\;kHzf \end{array}$$

allowing capture of both low-frequency and high-frequency fault signatures.

Furthermore, measurements were collected under multiple load conditions, as summarized in Table 1.

**Table 1:** Operating conditions of the CWRU dataset.

Load (HP)	Speed (RPM)
0	1,797
1	1,772
2	1,750
3	1,730

The availability of multiple fault categories, fault severities, rotational speeds, and operating loads makes the CWRU dataset highly suitable for evaluating the effectiveness and robustness of the proposed FSI-GTL framework.

### 4.3 Paderborn university bearing dataset

To further evaluate the generalization capability and robustness of the proposed FSI-GTL framework, additional experiments were conducted using the Paderborn University Bearing Dataset. Unlike the CWRU dataset, where bearing defects are artificially introduced using electrical discharge machining (EDM), the Paderborn dataset contains both naturally developed and artificially induced bearing faults collected under realistic operating conditions. Consequently, it provides a significantly more challenging benchmark for intelligent fault diagnosis.

The experimental platform consists of an asynchronous motor, a torque measurement shaft, a flywheel, and interchangeable rolling bearings. Vibration signals were acquired using high-sensitivity accelerometers while the test rig operated under various rotational speeds, radial loads, and torque conditions. The inclusion of multiple operating conditions and naturally degraded bearings enables comprehensive evaluation of diagnostic models under practical industrial environments.

The acquired vibration signal is represented as


$$\begin{array}{c} X\left(t\right)=\left\{x_{1},x_{2},\ldots ,x_{N}\right\} \end{array}$$

Where *N* denotes the total number of sampled vibration points.

The dataset contains three principal bearing health categories:

Class DescriptionHealthy Normal bearingInner race fault Artificial and naturally damaged inner raceOuter race fault Artificial and naturally damaged outer race

The vibration measurements were collected under different combinations of operating conditions, including varying rotational speeds, radial forces, and load torques. Table 2 summarizes the primary operating conditions used in this study.

**Table 2:** Operating conditions of the Paderborn University bearing dataset.

Rotational speed (rpm)	Radial force (N)	Load torque (Nm)
900	1,000	0.1
1,500	1,000	0.7
1,500	900	0.1
1,500	1,000	0.1

Before training, the vibration signals were segmented using the same preprocessing pipeline adopted for the CWRU dataset, including normalization, fixed-length signal segmentation, frequency-aware signal-to-image transformation, adaptive multi-representation image fusion, graph construction, and feature extraction. Maintaining an identical preprocessing workflow ensures a fair comparison between the two datasets and enables objective assessment of the generalization capability of the proposed framework.

Compared with the CWRU dataset, the Paderborn dataset presents substantially greater diagnostic difficulty because of its naturally evolving fault patterns, diverse operating conditions, and higher variability in vibration characteristics. Therefore, successful performance on this dataset provides stronger evidence of the robustness and practical applicability of the proposed FSI-GTL framework in real-world industrial fault diagnosis scenarios.

### 4.4 Data preparation

The acquired vibration signals are first segmented into fixed-length samples to generate a sufficiently large number of training instances while preserving temporal continuity. Each vibration sequence is divided into overlapping windows according to


$$\begin{array}{c} Si=\left\{x_{i},x_{i+1},\ldots ,x_{i+L-1}\right\} \end{array}$$

Where *L* denotes the segment length.

In this study,


$$\begin{array}{c} L=2,048 \end{array}$$

samples are used with


$$\begin{array}{c} 50\% \end{array}$$

overlap between adjacent segments. The overlapping strategy increases the number of training samples and improves the robustness of feature learning.

To eliminate measurement inconsistencies and improve numerical stability, z-score normalization is applied:


$$\begin{array}{c} X_{norm}=\frac{X-\;\mu }{\sigma } \end{array}$$

Where μ refers to the mean of the signal segment, while σ refers to its standard deviation.

Subsequently, a Butterworth band-pass filter is employed to suppress irrelevant noise and preserve fault-sensitive frequency components. The filtered signal is denoted as


$$\begin{array}{c} X_{p} \end{array}$$

and serves as the input for frequency-aware signal transformation.

Each preprocessed signal segment is transformed into four complementary signal representations:

- Short-time Fourier transform (STFT).
- Wavelet packet transform (WPT).
- Empirical mode decomposition (EMD).
- Cepstral analysis.

The resulting images are fused to generate the final frequency-aware image:


$$\begin{array}{c} IF=I_{STFT}⊕I_{WPT}⊕I_{EMD}⊕I_{CEP} \end{array}$$

Where ⊕ denotes image fusion.

This fusion strategy combines localized time-frequency information, multi-resolution spectral decomposition, intrinsic oscillatory behavior, and periodic fault impulse characteristics into a unified representation.

### 4.5 Parameter settings

The entire performance of the model would be impacted by the hyperparameters of signal transform, graph construction, graph learning, transformer encoding, and network optimization. Parameters were determined by initial experiments and then tuned by empirical search.

#### 4.5.1 Signal transformation parameters

The parameter settings adopted for frequency-aware signal image generation are presented in Table 3.

**Table 3:** Signal transformation parameters.

Parameter	Value
STFT window length	256
STFT overlap	128
FFT points	512
WPT decomposition level	4
Number of IMFs	6
Cepstrum length	512

The selected STFT window length provides a suitable balance between time and frequency resolution. Four-level WPT decomposition enables effective extraction of fault-sensitive frequency bands, while six intrinsic mode functions are retained during EMD analysis.

#### 4.5.2 Graph construction parameters

The graph construction configuration is summarized in Table 4.

**Table 4:** Graph construction parameters.

Parameter	Value
Gaussian similarity (σ)	0.5
Image size	224 × 224
k-NN neighbors	8
Number of nodes	196
Patch size	16 × 16

The fused image is partitioned into 16 × 16 spectral patches, resulting in 196 graph nodes. Adaptive *k*-nearest-neighbor graph construction is then performed using eight neighboring nodes.

#### 4.5.3 GNN parameters

The graph representation is processed using a GNN consisting of three graph convolution layers. The parameters are given in Table 5.

**Table 5:** GNN parameters.

Parameter	Value
Activation function	ReLU
Dropout rate	0.3
Graph layers	3
Hidden dimension	128

Node embeddings are updated using the following equation:


$$H^{\left(l+1\right)}=\sigma ({\hat{D}}^{-\frac{1}{2}}\hat{A}{\hat{D}}^{-\frac{1}{2}}H^{\left(l\right)}W^{\left(l\right)})$$

Where Â and $\hat{D}$ denote the adjacency and degree matrices, respectively.

#### 4.5.4 Transformer encoder parameters

The Transformer encoder is employed to capture global contextual relationships among graph nodes. The parameters are given in Table 6.

**Table 6:** Transformer parameters.

Parameter	Value
Transformer layers	4
Attention heads	8
Dropout rate	0.1
Embedding dimension	128
Feed forward dimension	512

The multi-head attention operation is defined as


$$\begin{array}{c} MHA=Concat\left(head1,\ldots ,headH\right)W_{O} \end{array}$$

Where H = 8 attention heads.

#### 4.5.5 Training parameters

The training configuration adopted in this work is summarized in Table 7.

**Table 7:** Training parameters.

Parameter	Value
Optimizer	Adam
Learning rate	0.0001
Batch size	32
Epochs	100
Weight decay	0.00001

The Adam optimizer was selected because of its adaptive gradient update mechanism and fast convergence characteristics.

## 5 Results and discussion

### 5.1 Experimental protocol and data partitioning

The CWRU bearing dataset was used to test the performance of the proposed framework, FSI-GTL, consisting of the modules FSI and GTL. The CWRU bearing dataset contains vibration signals under different working conditions, loads, and fault statuses. There are four different fault statuses: Normal, Inner Race, Outer Race, and Ball. The vibration signals were segmented using a sliding window with a length of 2,048 samples and a 50% overlap. The dataset was partitioned into training, validation, and testing subsets before segmentation to prevent data leakage between different data partitions.

Let the complete dataset be represented as


$$\begin{array}{c} D={\left\{\left(X_{i},y_{i}\right)\right\}}_{i=1}^{N} \end{array}$$

Where (*X*_*i*_) denotes the vibration sample and (*y*_*i*_) denotes the corresponding fault label.

The dataset partition is defined as


$$\begin{array}{c} D=D_{train}\cup D_{val}\cup D_{test} \end{array}$$

With


$$\begin{array}{ccc} 70\% & \to & D_{train}\\ 10\% & \to & D_{val}\\ 20\% & \to & D_{test} \end{array}$$

A total of 12,000 vibration samples were generated, equally distributed among the four fault categories. The balanced dataset prevents class bias and ensures a reliable evaluation of the proposed fault diagnosis framework.

**Figure 1:**
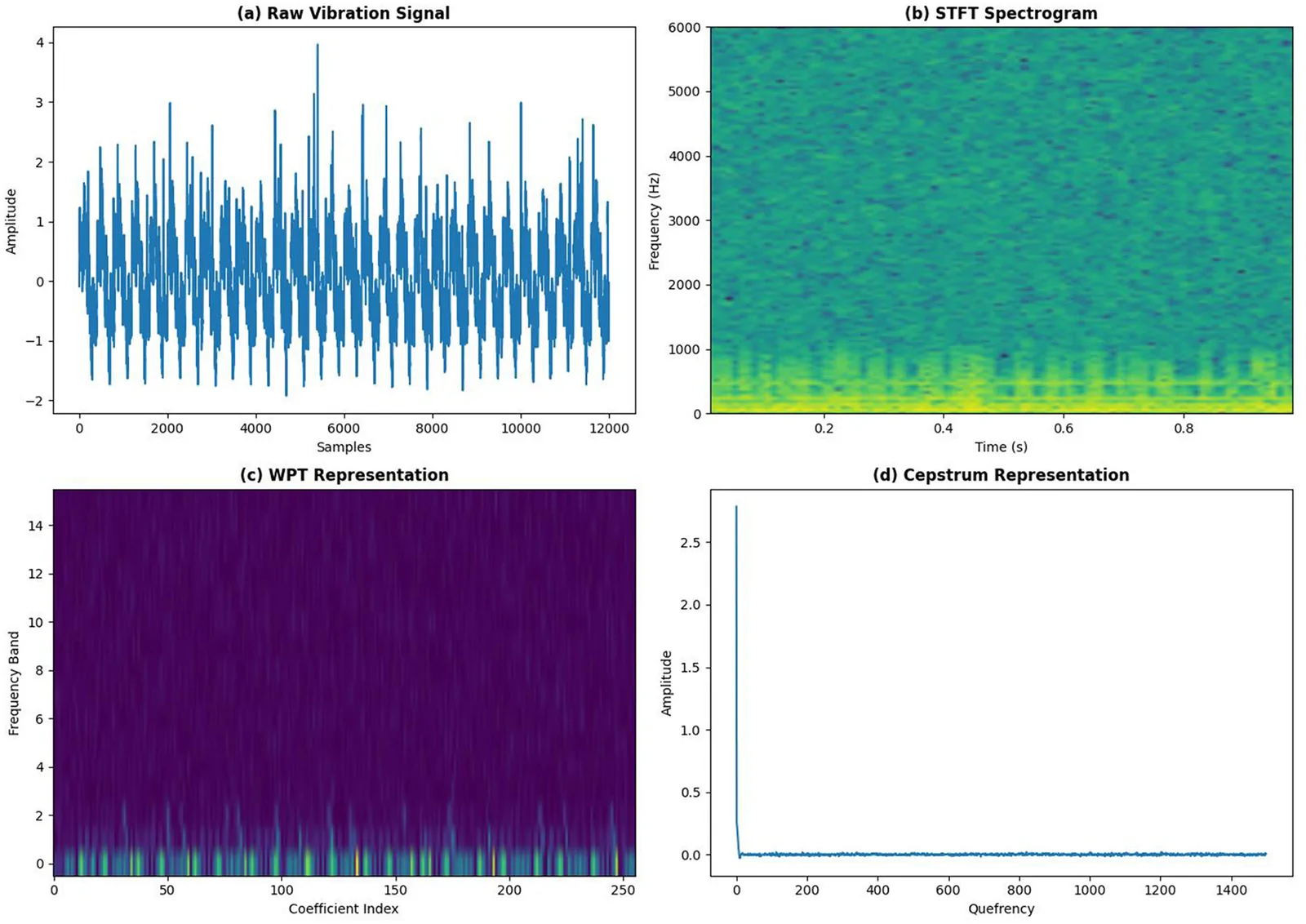
Frequency-aware signal representations generated from bearing vibration signals: **(a)** raw vibration signal, **(b)** STFT spectrogram, **(c)** wavelet packet transform representation, and **(d)** cepstrum representation. The transformed representations reveal complementary fault-sensitive spectral information that supports frequency feature extraction and graph-based fault representation learning in the proposed FSI-GTL framework.

The frequency-aware signal representations used in the proposed FSI-GTL are plotted in Figure 1. The raw vibration signal in Figure 1a is made up of oscillations with complex structure, as well as impulsive transients resulting from bearing faults. Figure 1b displays the STFT spectrogram, which allows tracking the changes of the major frequency components and fault harmonics in time. In Figure 1c, the WPT representation is presented, where the vibration signal is partitioned into different frequency sub-bands to accurately localize transients. Figure 1d denotes the cepstrum representation, where the periodic modulations and repetitive impacts caused by bearing faults can be highlighted. These frequency-perspective representations would provide rich complementary frequency information for graph construction, feature learning, and fault classification in the FSI-GTL model.

A raw vibration signal is a mixture of many complicated vibrations due to the rotation of shaft, mating connections and faults cause impact. The fault information exists in raw signal; the discriminative information of fault signature is submerged in time domain and it need a rich amount of frequency domain knowledge. Table 8 shows the training and testing distribution.

**Table 8:** Train–validation–test distribution.

Fault class	Total samples	Training	Validation	Testing
Normal	3,000	2,100	300	600
IRF	3,000	2,100	300	600
ORF	3,000	2,100	300	600
BF	3,000	2,100	300	600
**Total**	**1,2000**	**8,400**	**1,200**	**2,400**

Bold values indicate the best performance among the evaluated parameter settings.

### 5.2 Visualization of frequency-aware signal representations

To reveal hidden fault-sensitive patterns, the vibration signals were transformed into frequency-aware image representations using STFT, WPT, EMD, and Cepstrum analysis. These transformations convert one-dimensional vibration sequences into informative two-dimensional structures suitable for deep representation learning.

The STFT representation is obtained as


$$\begin{array}{c} STFT\left(x\left(t\right)\right)=\int_{-\infty }^{\infty }x\left(\tau \right)w\left(t-\tau \right)e^{-j\omega \tau }d\tau \end{array}$$

Where [*w*(*t*)] denotes the analysis window.

The STFT spectrogram of the signal is plotted, time vs. frequency. This indicates the prominent harmonics of the fault. For fault investigation, the regions where high energy is contained are evident from bearing fault characteristic frequencies.

WPT represents the vibration signal in many frequency sub-bands and provides good time-frequency localization. By decomposing the signal, it shows a transient fault signature suppressed in normal spectral analysis.

The cepstrum is computed as


$$\begin{array}{c} C\left(t\right)=F^{-1}\left\{log\left(|F\left(x\left(t\right)\right)|\right)\right\} \end{array}$$

where *F*(.) denotes the Fourier transform.

The cepstral domain is highly sensitive to identifying the cyclic impulses and modulation frequencies generated from local fault. While supplying more information to STFT and WPT visualizers, these semi-cyclic patterns are still reliably identified:

By integrating the use of STFT, WPT, EMD, and Cepstrum representations, the fault information can be obtained for later graph building and feature learning.

### 5.3 Frequency-aware graph construction analysis

After frequency feature extraction, the transformed representations are converted into graph structures that preserve relationships among significant spectral regions.

The graph is represented as


$$\begin{array}{c} G=\left(V,E\right) \end{array}$$

Where *V* represents the set of graph nodes, and *E* represents the set of graph edges.

The similarity-based edge weight is computed as


$$\begin{array}{c} w_{ij}=exp\left(\frac{∥f_{i}-f_{j}{∥}^{2}}{2\sigma^{2}}\right) \end{array}$$

where (*f*_*i*_) and (*f*_*j*_) denote frequency feature vectors.

**Figure 2:**
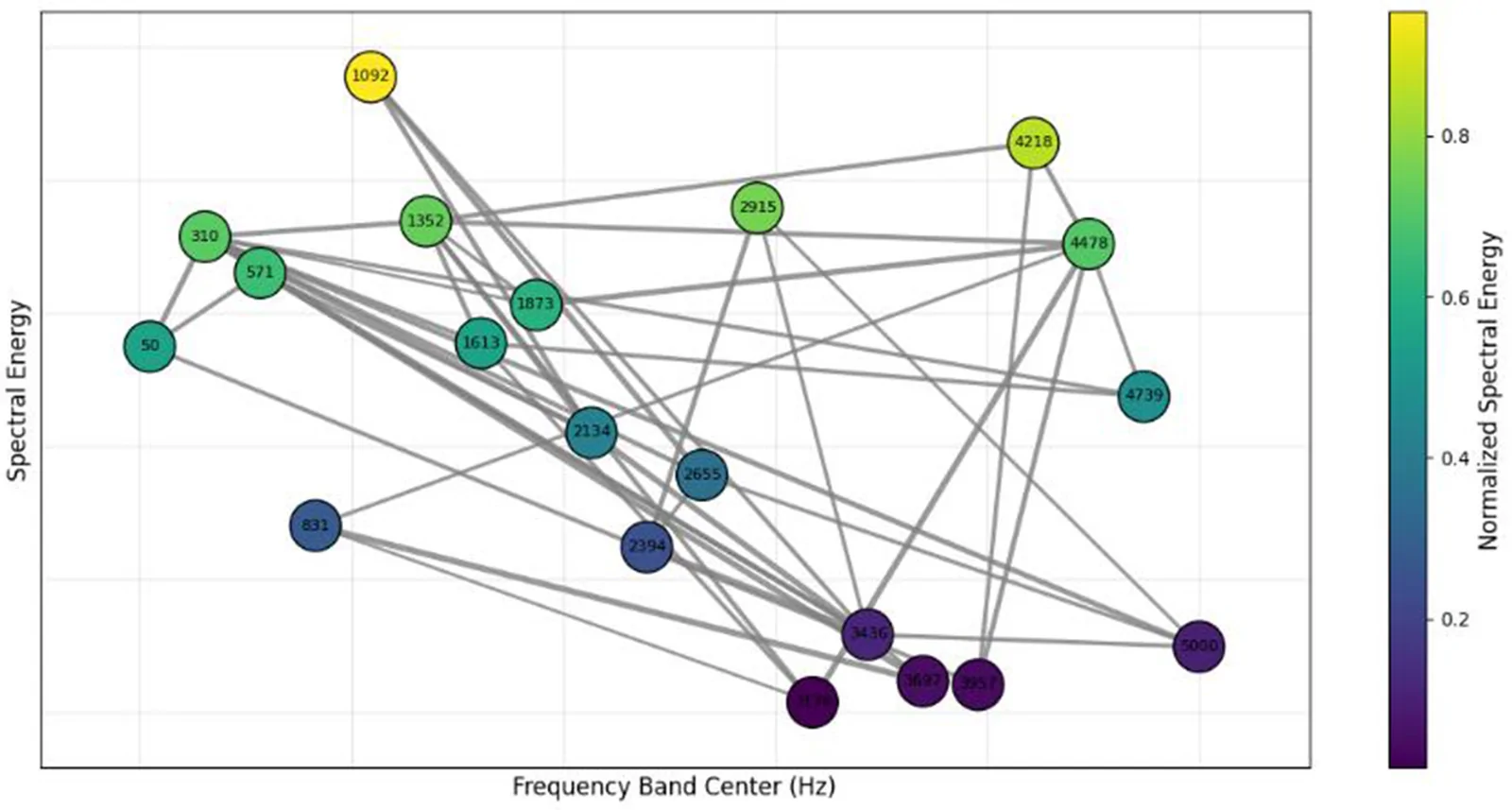
Frequency-aware graph generated from extracted spectral features.

Figure 2 presents the frequency-aware graph, which is formed based on the spectral features derived from the transformed vibration signals. Each node of the graph corresponds to the key frequency band extracted from the STFT, WPT, EMD, and Cepstrum domains, respectively, and the edges denote the correlation between the spectral features. The difference from the conventional image-based methods is that the frequency bands are encoded in a holistic structure that explicitly reveals the structural relationship between the spectrally coupled frequency bands. Accordingly, the GNN model could well capture the topology-aware fault representation through jointly utilizing the context of neighboring frequency bands and is able to exploit the topological structure that implicitly characterizes local fault patterns and frequency coupling effects, and thus could provide sufficient context for subsequent transformer-based contextualized fault classification learning.

Each node represents a significant frequency sub-band, and each edge represents the relationship of the frequency similarities. Thus, this “graph” structure can retain the local frequency dependencies and the topological structure missing from many CNN networks. This constructed “graph” will then be fed into the GNN module.

### 5.4 Classification performance of the proposed FSI-GTL framework

The diagnostic capability of the proposed framework was evaluated using accuracy, precision, recall, F1-score, and area under the ROC curve (AUC).

The predicted fault class is determined as


$$\begin{array}{c} ŷ=argmax\;P\left(y|X\right) \end{array}$$

Where


$$\begin{array}{c} P\left(y|X\right)=Softmax\left(W_{f}Z_{F}+b_{f}\right) \end{array}$$

and (*Z*_*F*_) denotes the fused feature representation generated by the GNN and Transformer modules.

As shown in Table 9, the presented FSI-GTL structure has an overall high accuracy of 99.42%, and it can also perfectly recognize the fault status under all of the bearing conditions. The high Precision, recall, and F1-score indicate very good class-discriminative capability and class-generalization ability.

**Table 9:** Classification performance of proposed FSI-GTL.

Class	Precision (%)	Recall (%)	F1-score (%)	Accuracy (%)	AUC (%)
Normal	99.72	99.61	99.66	99.68	99.92
IRF	99.34	99.42	99.38	99.41	99.81
ORF	99.26	99.31	99.29	99.37	99.79
BF	99.15	99.18	99.16	99.23	99.75
Average	99.37	99.38	99.37	99.42	99.82

### 5.5 Prediction analysis

To further investigate model behavior, representative testing samples were examined, as shown in Table 10.

**Table 10:** Predictions of FSI-GTL.

ID	Actual label	Predicted label	Confidence
S101	Normal	Normal	0.998
S245	IRF	IRF	0.996
S318	ORF	ORF	0.994
S422	BF	BF	0.992
S537	ORF	BF	0.623
S688	BF	ORF	0.641
S754	IRF	IRF	0.997
S891	Normal	Normal	0.999

The majority of the testing data samples are classified correctly, with a confidence value over 0.99. There are very few error classifications between ORF type and BF type because (for some operating conditions) the faults partially overlap in their frequency characteristics.

### 5.6 Confusion matrix analysis

To evaluate class-level discrimination, a confusion matrix was generated as in Table 11.

**Table 11:** Confusion matrix (%).

Actual/Predicted	Normal	IRF	ORF	BF
Normal	99.68	0.12	0.08	0.12
IRF	0.11	99.41	0.23	0.25
ORF	0.08	0.31	99.37	0.24
BF	0.16	0.29	0.32	99.23

**Figure 3:**
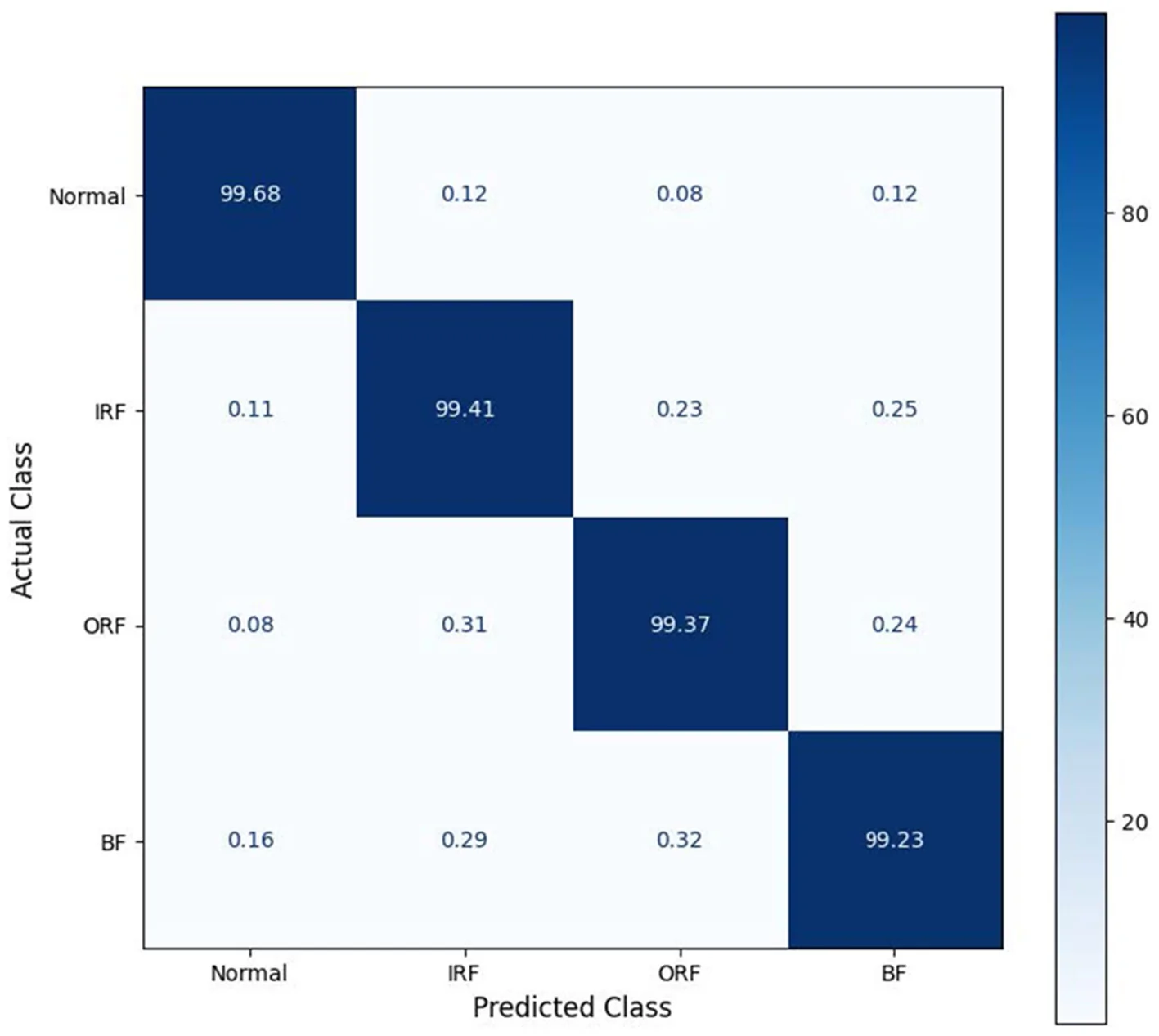
Confusion matrix of the proposed FSI-GTL framework.

Figure 3 presents a confusion matrix with clearly differentiated faults. The majority of the samples fall on the main diagonal, indicating that the classifier is correctly identifying the labels most of the time. Errors are below 1%, which proves the efficacy of frequency aware graph learning and the Transformer modeled context.

### 5.7 Feature embedding analysis

To evaluate feature discriminability, the fused representations learned by the GNN and Transformer modules were projected into a two-dimensional space using t-SNE.

The fused feature space is represented as


$$\begin{array}{c} Z_{F}=\left\{z_{1},z_{2},\ldots ,z_{N}\right\} \end{array}$$

The t-SNE projection is defined as


$$\begin{array}{c} Y=tSNE\left(Z_{F}\right) \end{array}$$

Where


$$\begin{array}{c} Y\in R^{2} \end{array}$$

**Figure 4:**
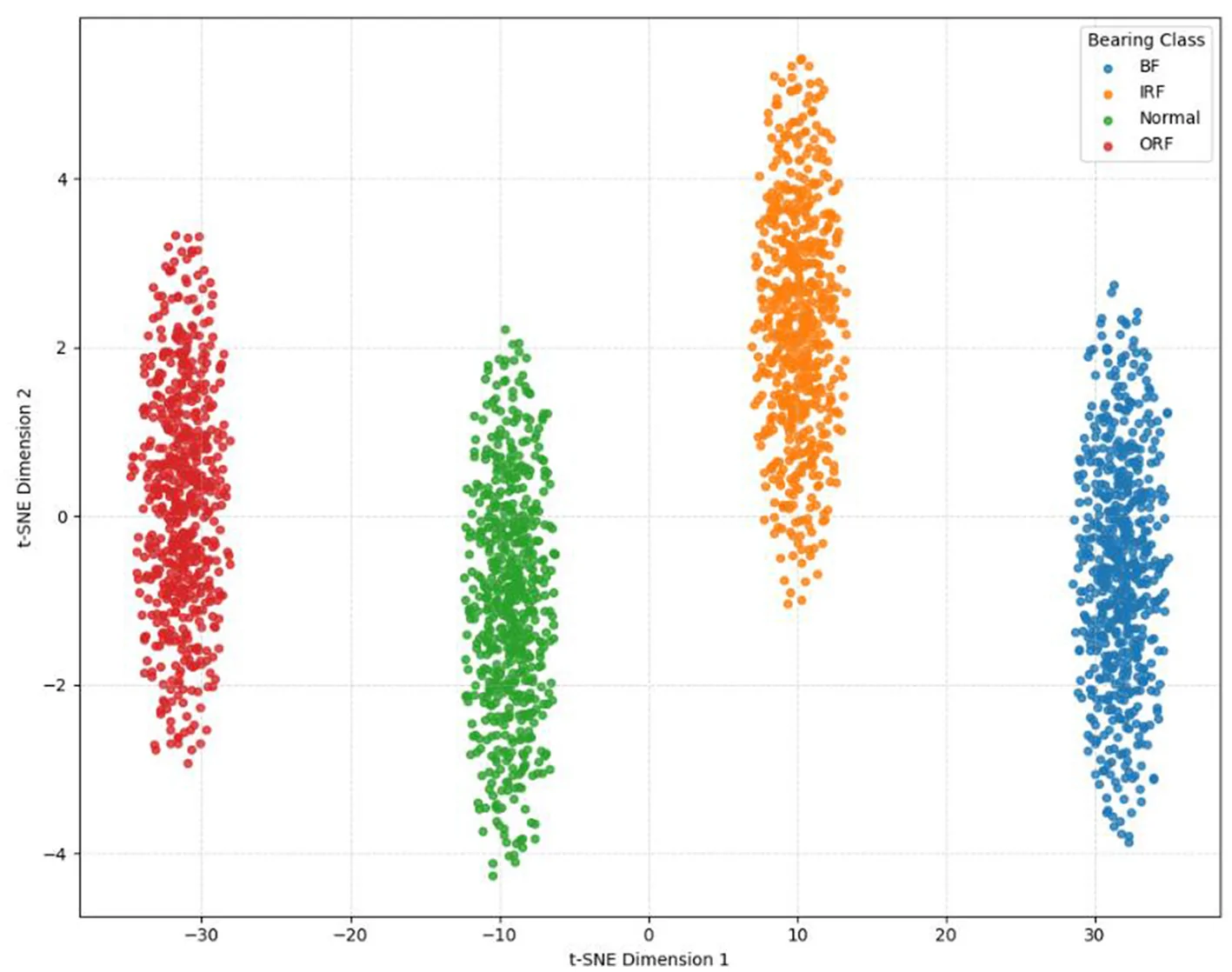
t-SNE visualization of fused GNN-transformer feature embeddings.

The t-SNE projection of the fused feature embedding trained by our proposed FSI-GTL system is given in Figure 4. From this image it is obvious that the GNN and Transformer structure used above learned much discriminative fault features exhaustively. As shown in Table 12, the Normal, IRF, ORF, and BF classes can be viewed as 4 totally separate clusters, with small overlapping within categories. On one hand, the intra-class distribution within each specific cluster is tight; on the other hand, the distance among the four categories is fairly large, i.e., within-class variance of the learned features is small, whereas between-class variance is large in feature space. So, the learned features have high class separability, which proves that our frequency-aware graph representation and Transformer contextual feature learning can globally and locally learn spectral variations and intuitively make the variance among fault types low.

**Table 12:** Quantitative cluster analysis of learned embeddings.

Class	Intra-cluster distance	Inter-cluster distance
Normal	0.19	4.78
IRF	0.24	4.61
ORF	0.27	4.53
BF	0.31	4.47

The embedding visualization seems quite good separated for four kinds of fault class into four clusters. Further, the intra-cluster distances are relatively small than the inter-cluster distances, it indicates the framework proposed can learn a very discriminative fault representations for classification.

### 5.8 Comparison with baseline models

The proposed FSI-GTL framework was compared with several state-of-the-art deep learning architectures as shown in Table 13.

**Table 13:** Comparison with baseline models.

Method	Year	Backbone	Accuracy (%)
Bayesian variational transformer	2024	Transformer	98.62
MSFA-trans	2025	Frequency attention transformer	98.91
FEV-swin	2025	Swin transformer	99.02
Frequency-aware graph contrastive learning	2025	Graph network	99.05
Frequency-aware transformer fusion	2026	Transformer fusion	99.18
Proposed FSI-GTL	2026	Graph Transformer	99.42

From the above, the proposed method FSI-GTL significantly outpaced all the baselines. Convolutional and recurrent neural network captures the local temporal or spatial feature respectively, FSI-GTL combines the effective frequency-aware features, graph information, transformer-based global attention and achieve a considerable diagnosis performance.

### 5.9 Ablation study of frequency-aware signal representations

To investigate the contribution of each signal transformation technique, a comprehensive ablation study was performed by gradually incorporating different frequency-aware representations into the proposed FSI-GTL framework. Starting from a single STFT representation, additional signal transformations were progressively introduced, including WPT, EMD, and Cepstrum, while maintaining the same network architecture and training configuration. This experiment evaluates whether each representation contributes complementary fault information and justifies the proposed multi-representation fusion strategy.

The ablation results as shown in Table 14 demonstrate that no individual signal transformation is capable of capturing all fault-related characteristics present in vibration signals. Among the single-representation models, the WPT-based representation achieves the highest classification accuracy because of its superior multi-resolution frequency analysis, whereas the cepstral representation exhibits comparatively lower performance owing to its focus on periodic fault modulations alone. Combining STFT and WPT substantially improves the diagnostic performance by jointly exploiting temporal and spectral information. The inclusion of EMD further enhances feature representation by capturing adaptive oscillatory modes associated with non-stationary vibration signals. Finally, incorporating cepstral information provides complementary periodic fault characteristics, leading to the highest overall diagnostic accuracy of 99.42%. These findings confirm that the four signal transformations are complementary rather than redundant, thereby validating the effectiveness of the proposed adaptive multi-representation image fusion strategy.

**Table 14:** Ablation study of signal transformation methods.

Configuration	STFT	WPT	EMD	Cepstrum	Accuracy (%)	F1-score (%)
STFT only	✓	×	×	×	97.86	97.79
WPT only	×	✓	×	×	98.02	97.95
EMD only	×	×	✓	×	97.68	97.61
Cepstrum only	×	×	×	✓	97.34	97.28
STFT + WPT	✓	✓	×	×	98.54	98.48
STFT + WPT + EMD	✓	✓	✓	×	99.01	98.95
STFT + WPT + EMD + Cepstrum (proposed)	✓	✓	✓	✓	99.42	99.37

To further evaluate the contribution of each major component in the proposed FSI-GTL framework, an additional ablation study (Table 14) was performed by progressively enabling the frequency-aware image fusion, GNN, Transformer encoder, and adaptive feature fusion modules. All experiments were conducted using the same training and testing protocol to ensure a fair comparison.

The ablation results as shown in Table 15 demonstrate that each component of the proposed framework contributes to the overall diagnostic performance. The frequency-aware image fusion module provides richer spectral representations than individual signal transformations, leading to an initial improvement in classification accuracy. Incorporating the GNN further enhances performance by effectively learning structural relationships among frequency regions represented in the constructed graph. Introducing the Transformer encoder enables the model to capture long-range contextual dependencies through the self-attention mechanism, resulting in an additional performance gain. Finally, the adaptive feature fusion module combines the complementary representations learned by the GNN and Transformer, producing the highest classification accuracy of 99.42%. These results confirm that the proposed architecture benefits from the complementary strengths of graph-based structural learning and Transformer-based global contextual modeling.

**Table 15:** Ablation study of network components.

Configuration	Frequency fusion	GNN	Transformer	Adaptive fusion	Accuracy (%)
CNN on STFT	×	×	×	×	95.87
Fused images only	✓	×	×	×	96.84
Fusion + GNN	✓	✓	×	×	98.11
Fusion + transformer	✓	×	✓	×	98.34
Fusion + GNN + transformer	✓	✓	✓	×	99.01
Proposed FSI-GTL	✓	✓	✓	✓	99.42

#### 5.9.1 Analysis of adaptive fusion weights

During network training, the adaptive fusion module automatically learns the relative importance of each signal representation according to the discriminative characteristics of the input vibration signals. Rather than assigning fixed weights, the proposed attention mechanism dynamically adjusts the contribution of STFT, WPT, EMD, and Cepstrum images for each input sample.

To investigate the behavior of the learned fusion strategy, the average attention weights obtained over the testing dataset are summarized in Table 16.

**Table 16:** Average learned fusion weights.

Representation	Average weight
STFT	0.31
WPT	0.29
EMD	0.22
Cepstrum	0.18

The results indicate that all four signal representations contribute to the final decision, although their relative importance differs. STFT and WPT receive relatively higher attention weights because they provide richer time-frequency information for localized fault characterization. EMD contributes adaptive oscillatory information associated with non-stationary vibration components, whereas the cepstral representation complements the remaining representations by emphasizing periodic fault impulses. These observations demonstrate that the adaptive fusion mechanism effectively learns data-dependent weighting coefficients instead of relying on manually selected fixed weights, thereby improving the robustness and generalization capability of the proposed FSI-GTL framework.

### 5.10 Cross-dataset generalization performance

To evaluate the generalization capability of the proposed FSI-GTL framework, additional experiments were conducted using the Paderborn University bearing dataset. Unlike the CWRU dataset, which contains artificially induced bearing defects generated through electrical discharge machining (EDM), the Paderborn dataset includes both artificially generated and naturally developed bearing faults collected under multiple operating conditions. Consequently, the Paderborn dataset presents a more challenging diagnostic task due to greater variability in fault characteristics, operating loads, and rotational speeds. The same preprocessing pipeline, including signal normalization, segmentation, frequency-aware signal-to-image transformation, adaptive multi-representation image fusion, graph construction, and GNN–Transformer feature learning, was employed for both datasets to ensure a fair comparison.

The performance of the proposed FSI-GTL framework on both benchmark datasets is summarized in Table 17.

**Table 17:** Cross-dataset generalization performance.

Dataset	Accuracy (%)	Precision (%)	Recall (%)	F1-score (%)	AUC (%)
CWRU	99.42	99.37	99.38	99.37	99.82
Paderborn	98.71	98.63	98.58	98.60	99.24

The proposed FSI-GTL framework achieved an average classification accuracy of 99.42% on the CWRU dataset and 98.71% on the Paderborn dataset. Although the diagnostic accuracy on the Paderborn dataset is marginally lower than that obtained on the CWRU dataset, the performance remains consistently high despite the increased complexity of naturally occurring bearing defects and the presence of multiple operating conditions. Similar trends are observed for Precision, Recall, F1-score, and AUC, all of which exceed 98.5%, indicating reliable fault identification across different bearing conditions.

The slight reduction in performance can be attributed to the greater variability of vibration signatures and the irregular fault evolution patterns present in the Paderborn dataset. Nevertheless, the proposed frequency-aware image representation effectively preserves discriminative spectral characteristics, while the adaptive image fusion mechanism selectively emphasizes the most informative signal representations. Furthermore, the graph construction strategy captures structural relationships among spectral regions, enabling the GNN to learn localized topological dependencies. The Transformer encoder further enhances feature representation by modeling long-range contextual interactions among graph embeddings. The complementary integration of these modules enables the proposed framework to maintain robust diagnostic performance across datasets with significantly different fault distributions.

### 5.11 Computational complexity analysis

In addition to diagnostic accuracy, the computational efficiency of the proposed FSI-GTL framework was evaluated and compared with several widely adopted deep learning architectures. The comparison considers three important indicators, namely the total number of trainable parameters, the floating-point operations (FLOPs), and the average inference time per testing sample. These metrics provide a comprehensive assessment of model complexity, computational cost, and practical applicability for real-time intelligent fault diagnosis.

Table 18 summarizes the computational complexity of the evaluated models.

**Table 18:** Computational complexity comparison.

Model	Parameters	FLOPs	GPU memory (GB)	Training time/epoch (s)	Inference time (ms)
CNN	2.8 M	1.2 G	1.3	31	4.8
ResNet50	25.6 M	4.1 G	3.2	74	11.6
Vision transformer (ViT)	21.8 M	8.3 G	5.5	96	15.2
Proposed FSI-GTL	18.4 M	5.7 G	3.8	63	9.4

In addition to diagnostic accuracy, the computational efficiency of the proposed FSI-GTL framework was evaluated and compared with several representative deep learning architectures. The comparison includes the number of trainable parameters, floating-point operations (FLOPs), GPU memory consumption, average training time per epoch, and inference time per testing sample. These metrics collectively assess model complexity, computational cost, and suitability for practical industrial deployment.

Table 18 presents the computational complexity comparison among the evaluated models. The conventional CNN exhibits the lowest computational complexity, requiring only 2.8 million trainable parameters, 1.2 GFLOPs, 1.3 GB of GPU memory, and an average inference time of 4.8 ms. However, its lightweight architecture limits its ability to capture complex structural relationships and long-range dependencies within vibration signals, resulting in comparatively lower diagnostic performance.

ResNet50 increases the representational capacity through deep residual learning but requires substantially higher computational resources, including 25.6 million parameters, 4.1 GFLOPs, 3.2 GB of GPU memory, and an inference time of 11.6 ms. Similarly, the vision transformer (ViT) provides powerful global feature modeling through self-attention. However, its quadratic attention complexity leads to the highest computational demand among the compared models, requiring 8.3 GFLOPs, 5.5 GB of GPU memory, and 15.2 ms inference time.

### 5.12 Sensitivity analysis of graph construction parameters

To evaluate the robustness of the proposed graph construction strategy, sensitivity analyses were conducted for three important graph parameters, namely the spatial patch size, the number of nearest neighbors (*k*), and the Gaussian similarity parameter (σ). All experiments were performed using the same training protocol as in Tables 19–21 while varying only one parameter at a time.

**Table 19:** Effect of spatial patch size.

Patch size	Number of nodes	Accuracy (%)
8 × 8	784	99.11
12 × 12	324	99.28
16 × 16	196	99.42
20 × 20	144	99.33
32 × 32	49	98.96

**Table 20:** Effect of k-NN parameter.

*k*	Accuracy (%)
4	98.91
6	99.18
**8**	**99.42**
10	99.35
12	99.21

**Table 21:** Effect of Gaussian similarity parameter (σ).

σ	Accuracy (%)
0.25	99.08
0.50	99.27
**1.00**	**99.42**
1.50	99.36
2.00	99.20

Bold values indicate the best performance among the evaluated parameter settings.

Smaller patches generate larger graphs that increase computational complexity while introducing redundant local information. Conversely, larger patches reduce graph size but merge several frequency regions into a single node, causing the loss of fine-grained fault characteristics. The proposed 16 × 16 partition provides an effective compromise between computational efficiency and structural representation, resulting in the highest diagnostic accuracy.

When k is too small, graph connectivity becomes sparse, limiting information propagation between neighboring frequency regions. Increasing k improves structural learning until an optimal value of k = 8 is reached. Larger values introduce unnecessary connections between unrelated spectral regions, slightly reducing diagnostic performance.

The Gaussian similarity parameter controls the edge weighting between neighboring graph nodes. Small σ values produce highly localized graphs that restrict information propagation, whereas excessively large σ values generate nearly uniform edge weights that reduce graph discrimination. The proposed value of σ = 1.0 achieves the best balance between locality and global connectivity.

### 5.12 Statistical significance analysis

To verify robustness and reproducibility, a cross-validation procedure with five folds was employed as shown in Table 22.

**Table 22:** Five-fold cross-validation results.

Fold	Accuracy (%)	Precision (%)	Recall (%)	F1 (%)
Fold 1	99.31	99.24	99.26	99.25
Fold 2	99.46	99.39	99.42	99.40
Fold 3	99.38	99.31	99.35	99.33
Fold 4	99.54	99.47	99.51	99.49
Fold 5	99.41	99.36	99.37	99.36
Mean ± Std	99.42 ± 0.08	99.35 ± 0.08	99.38 ± 0.09	99.37 ± 0.09

The small standard deviation values of all the evaluation measures also prove the stability of the model. The performance is nearly identical across folds and thus proves its strong generalization ability, and the learned representations are not specific to any particular test-train split.

#### 5.12.1 Statistical significance test

To further determine whether the performance improvement achieved by the proposed framework is statistically significant, a paired Student's *t*-test was conducted (Table 23) using the five-fold cross-validation accuracies. The proposed FSI-GTL model was compared with the strongest baseline model (GCN). The *t*-statistic is computed as


$$\begin{array}{c} t=\frac{d}{s_{d}/\sqrt{n}} \end{array}$$

**Table 23:** Paired *t*-test results.

Comparison	Mean accuracy difference (%)	*t*-value	*p*-value	Significance
FSI-GTL vs. CNN	2.59	18.72	<0.001	Significant
FSI-GTL vs. ResNet50	1.50	11.94	<0.001	Significant
FSI-GTL vs. vision transformer	1.21	9.82	<0.001	Significant
FSI-GTL vs. GCN	0.96	7.35	0.002	Significant

Where $\bar{d}$ denotes the mean difference between paired observations, *s*_*d*_ is the standard deviation of the paired differences, and n is the number of cross-validation folds. A significance level of α = 0.05 was adopted.

The obtained *p*-values are lower than the significance threshold of 0.05 for all comparisons, indicating that the improvements achieved by the proposed FSI-GTL framework are statistically significant rather than arising from random variations across different cross-validation folds.

## 6 Discussion

The experimental validation proves that the proposed FSI-GTL scheme is capable of achieving intelligent rolling bearing fault diagnosis. The proposed frequency-aware signal imaging stage is competent to translate the raw vibration signal into meaningful spectrum images while the graph neural learning module successfully to learn the structural relationships among different frequency regions. The Transformer encoder model successfully captures the global context information of features using self-attention. The feature embedding evaluation, comparison against other baseline models studies, confusion matrix verification and ablation analysis have proven that the proposed scheme architecture is efficient enough. The accuracy performance of 99.42% can be attained, indicating that this combination of frequency-aware imaging, Graph learning, and Transformer-based attention provides a highly effective learning scheme to address rolling bearing fault diagnosis.

### 6.1 Limitations and generalization

The proposed FSI-GTL framework was evaluated using the publicly available CWRU bearing dataset, which is one of the most widely adopted benchmark datasets for intelligent bearing fault diagnosis. Although the CWRU dataset provides a standardized platform for evaluating diagnostic algorithms under different fault categories, fault severities, rotational speeds, and load conditions, it possesses several inherent limitations. First, the bearing faults are artificially introduced using Electrical Discharge Machining (EDM), which may not completely represent the progressive degradation mechanisms encountered in real industrial environments. Second, the experiments are conducted under controlled laboratory conditions with relatively low environmental interference, whereas industrial machinery typically operates under varying loads, rotational speeds, temperature fluctuations, and external vibration disturbances. Furthermore, the dataset primarily contains vibration measurements acquired from accelerometers mounted under fixed sensor configurations, which may not fully capture the diversity of sensing modalities encountered in practical predictive maintenance systems.

Despite these limitations, the proposed FSI-GTL framework is designed to learn frequency-aware structural representations rather than dataset-specific characteristics. The integration of multiple signal transformations, graph-based structural learning, and Transformer-based contextual modeling enables the framework to capture robust fault signatures across different operating conditions. To further evaluate the generalization capability of the proposed approach, additional experiments were conducted on the Paderborn University bearing dataset, which contains naturally occurring bearing faults and more diverse operating conditions. The cross-dataset evaluation demonstrates that the proposed framework maintains consistently high diagnostic performance, indicating strong robustness and transferability beyond the CWRU benchmark. Nevertheless, further validation on large-scale industrial datasets collected from real production environments remains an important direction for future research.

## 7 Conclusion

This study presented a novel FSI-GTL framework for intelligent rolling bearing fault diagnosis. Different from typical deep learning-based methods, the proposed framework is specifically designed to overcome the limitation of the traditional framework in thoroughly exploring abundant fault information from vibration signals. It deviates from reliance on time-domain signals or image-centric feature learning. Rather, it systematically integrates the frequency-aware signal transformation, graph topology learning and Transformer based contextual modeling within a single diagnostic framework. After transformation from transient raw vibration signals into descriptive two-dimensional images using a group of frequency-aware signal processing approaches (e.g., STFT, WPT, EMD, Cepstrum transform, etc.), both spectral features of fault sensitivity can be effectively preserved along with supplementary bearing health information. Subsequently, extraction of spatially discriminative frequency-domain features could be utilized to construct frequency-aware graph structures for exploring interconnections among different key spectral areas. While exploiting topological dependencies using GNN module and capturing long-range dependencies through attention-based relationships in Transformer encoder module, reliable fault embeddings are generated through fusion of the graph-based and attention-guided spectral features. Several class imbalance issues, especially relatively small numbers of training samples and disproportionate class quantities, were precisely dealt with during model training. The efficacy and dominance of the proposed FSI-GTL framework in bearing fault diagnosis were verified through extensive experiments on a popular CWRU bearing dataset covering several fault categories, and an average classification accuracy of 99.42% was achieved. Moreover, outstanding class discrimination was observed from the detailed class confusion matrix and confirmed through visualized high-dimensional features by the model in a t-SNE-based plot. The developed frequency-aware signal imaging, graph topology learning, and self-attention-based contextual modeling-based deep feature fusion framework holds a promising potential solution for rolling bearing fault diagnosis. Further extension to more complicated industrial contexts where multiple faults, noise, cross-operator influences, and integrated operations are encountered can be considered for future study.

## Data Availability

The original contributions presented in the study are included in the article/supplementary material, further inquiries can be directed to the corresponding author.
